# Beyond the physical: exploring the complexities of Women’s health after severe perineal trauma—a cross-sectional study on predictors of health-related quality of life in Sweden

**DOI:** 10.3389/fgwh.2026.1734365

**Published:** 2026-02-12

**Authors:** Katharina Tjernström, Inger Lindberg, Maria Wiklund, Margareta Persson

**Affiliations:** 1 Department of Nursing, Umeå University, Umeå, Sweden; 2 Department of Community Medicine and Rehabilitation, Section of Physiotherapy, Umeå University, Umeå, Sweden

**Keywords:** cross-sectional study, health-related quality of life, long-term, obstetric anal sphincter injury, patient-reported outcome measures, predictors, RAND-36, severe perineal trauma

## Abstract

**Introduction:**

Severe perineal trauma (SPT), defined as third- or fourth-degree lacerations during childbirth, is a known risk factor for adverse postpartum health-related quality of life (HRQoL). Although HRQoL may improve within six months postpartum, up to 30% of affected women in Sweden report long-term health problems beyond one year. While qualitative studies highlight the broad negative impact, quantitative findings remain inconclusive, particularly regarding the role of pelvic floor symptoms and the degree of SPT, underscoring the need for further research. The aim is to assess and compare HRQoL in a sample of women with SPT, and to identify predictors of physical and mental health at least 18 months postpartum.

**Methods:**

A nationwide cross-sectional study was conducted in Sweden using an online questionnaire to assess HRQoL via the validated RAND-36 instrument. Linear regression analysis was employed to explore associations.

**Results:**

Two hundred and twenty-one women with SPT and varying symptom bother from SPT, at least 18 months after the childbirth, responded. The study population exhibited worse-than-average RAND-36 scores across most dimensions (apart from physical functioning and pain) compared to normative data for women in Sweden. Further, the mean physical health score was significantly lower in our study sample (*M* = 70.7, *SD* = 22.1) compared to the reference population of women in Sweden (*M* = 73.63, *SD* = 29.45), *t* [*df* (degrees of freedom) 220] = −1.99, *p* = 0.047, Cohen's d = 0.13. The mean mental health score was significantly lower in our study sample (*M* = 63.2, *SD* = 21.4) compared to normative Swedish women (*M* = 71.7, *SD* = 27.15), *t* (*df* 220) = −5.90, *p* < 0.001, Cohen's d = 0.40. Health change over the past year remained relatively static with a slight trend towards improvement (mean 54.5; *SD* 21.6; *CI* 95% 51.6–57.4). Physical health was predicted by the extent of symptom bother, perceived work ability, educational attainment, and level of physical activity. Mental health was predicted by age, extent of symptom bother, and perceived work ability.

**Conclusions:**

These findings underscore the need for individualized, multidisciplinary care strategies that address both physical and psychological dimensions of recovery after sustaining SPT at childbirth. Future research should investigate the barriers and facilitators influencing HRQoL, to enhance HRQoL and support the reintegration of women with SPT into their social and professional spheres. A deeper understanding of the socioeconomic and occupational contexts of affected women is essential to promote more equitable health outcomes.

## 1 Introduction

Severe perineal trauma (SPT), defined as third- or fourth-degree perineal laceration during childbirth (1), can lead to long-term physical, psychological, and social consequences (2–8). SPT is a recognized risk factor for adverse postpartum quality of life (9). Its incidence ranges from 0.1%–5.2% in Europe (10, 11) and about 2%–3% in Nordic countries (12, 13). In Sweden, the 2023 incidence was 2.7% (13), with systematic postpartum follow-up via the National Perineal Laceration Register (14). Despite this, around 30% of Swedish women with SPT report complications after one year (14).

Health-related quality of life (HRQoL) (15) defines a specific dimension of overall quality of life that pertains to health. HRQoL includes both subjective perceptions and objective assessments of physical, psychological, and social well-being, forming a multifaceted construct. Furthermore, HRQoL evaluates the impact of health conditions, diseases, and treatments on an individual's life, considering personal experiences and actual functioning across various health domains, while also encompassing the values attributed to different health states (15). Although HRQoL appears to improve among women with SPT during the first six months (16), the long-term (beyond one year postpartum) implications of SPT on HRQoL remain insufficiently studied. SPT might entail substantial emotional distress, such as anxiety, loneliness, and shame, which negatively influences Women’s daily lives (2). Inconsistent support from healthcare professionals further exacerbates anxiety and stress after SPT (2, 17). Additionally, SPT significantly influences Women’s reproductive decisions, family planning (18), and professional lives (19). Besides mental health problems (2), SPT can lead to a variety of long-term physical morbidities, including pain (2), incontinence (3), defecation problems (4), vaginal prolapse (5), and sexual dysfunction (6). Despite this, research on HRQoL after SPT has predominantly focused on anal incontinence, with most studies indicating a significant negative impact on HRQoL (20–25). In this context, women with anal incontinence experience loss and grief while struggling with their complex social roles. They also find themselves caught between personal and professional silence or disclosure, employing strategies such as avoidance, denial, and compromise to regain normality (7, 21).

This study addresses the knowledge gap of whether other pelvic floor symptoms and the grade of SPT impact HRQoL (26–29) or not (16, 25, 30). Changes in HRQoL have been used to evaluate recommendations for subsequent birth mode after SPT (31–34) and various treatments, including surgical approaches (35–37) and physical therapy (38, 39). Qualitative research suggests a negative impact on Women’s daily lives, including limitations in social life, daily activities, motherhood, and sexual functioning, while quantitative studies are inconclusive due to methodological limitations (8). Thus, the aim is to assess and compare HRQoL in a sample of women with SPT, and to identify predictors of physical and mental health at least 18 months postpartum.

## 2 Materials and methods

### 2.1 Study design

The present study was designed as a national cross-sectional online survey study, which is part of a larger research initiative (17, 19, 40) aimed at exploring the long-term impacts of SPT on HRQoL, employment, and healthcare interactions.

### 2.2 Study context

In general, individuals aged 20–49 in Sweden report similar HRQoL (41), while notable declines in physical health are observed in older age groups, particularly among those aged 80 and above. Significant age-related differences are also evident in mental health; however, older age groups, excluding the oldest (those aged 80 and above), report higher energy and emotional well-being. Additionally, individuals with a university education exhibit significantly higher HRQoL compared to those with only mandatory education. Employees have the highest HRQoL, whereas individuals on sick leave have the lowest. Finally, men in Sweden exhibit significantly higher HRQoL than women (41).

Life expectancy for women in Sweden is high in global comparison (84.3 years), and the maternal mortality rate is four deaths per 100,000 live births (42). Parental leave of up to 480 days per child, as well as healthcare, is tax-funded (43, 44). Gender differences in health reflect global trends, with men experiencing higher mortality rates, while women demonstrate greater morbidity and utilize healthcare services more frequently (45). Women are predominantly on sick leave for psychiatric and musculoskeletal conditions and exhibit higher sickness rates than men (46). The employment rate for women stands at 72.8%, with a substantial proportion employed in the healthcare and education sectors (47).

In Sweden, sexual and reproductive health services are predominantly delivered within the primary care sector. In contrast, childbirth care is almost exclusively hospital-based, with home births and freestanding birth centers representing exceptionally uncommon alternatives. Midwives autonomously manage care during normal pregnancies and uncomplicated births, whereas obstetricians are consulted in complicated cases (48, 49). As late as 2025, national guidelines for pelvic floor dysfunction were implemented in Sweden (50).

### 2.3 Participants and recruitment

The study involved adult women (18 years or older) proficient in understanding and speaking Swedish who had experienced third- or fourth-degree perineal lacerations at least 18 months before the data collection, irrespective of vaginal birth mode. Participants were selected without regard to the existence or seriousness of self-reported SPT-related health problems, as well as whether they had undergone reconstructive surgery or not. Conversely, women were not eligible if they were still on full-time parental leave after SPT or pregnant and were excluded if they experienced a stillbirth or perinatal death of the baby within 28 days postpartum at the time of the SPT.

A digital poster was disseminated through various online channels focusing on perineal health and pelvic floor functioning, including social media platforms such as Facebook, Instagram, and TikTok, as well as blogs and interest groups. Additionally, our department's social media account on Facebook contributed to this effort. The poster outlined the research project and linked to the project's homepage, which provided detailed written information on the project, contact information for the research team, and a link to the Research Electronic Data Capture (REDCap)-based (51, 52) questionnaire for participation.

### 2.4 Data collection

**Figure 1:**
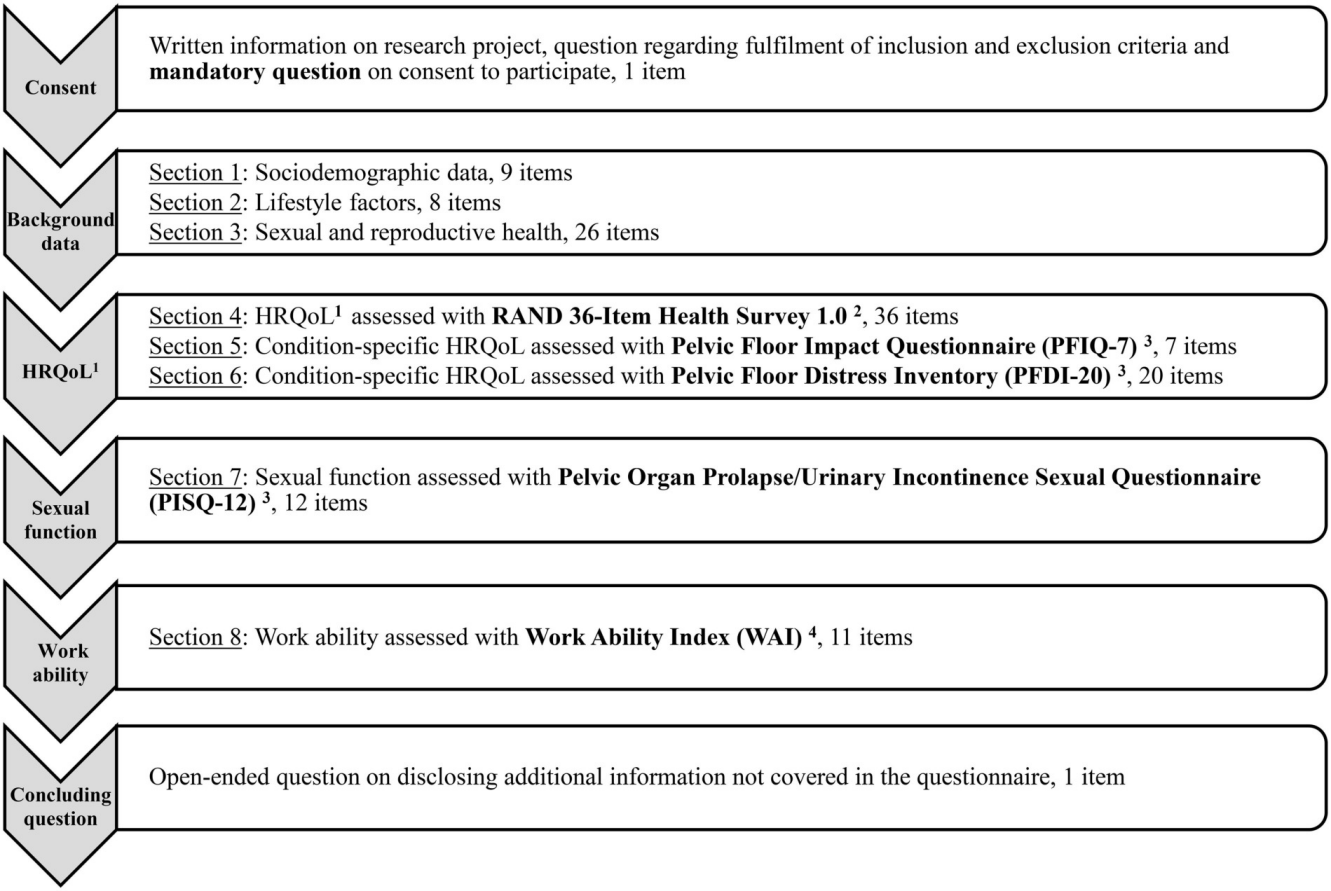
Overview of the questionnaire employed in the present study with consent being the only mandatory question. ^1^HRLQoL = health-related quality of life; ^2^Hays R, Sherbourne C, Mazel R. The Rand 36-item Health Survey 1.0. Health Econ. (1993) 2(3):217-27. doi: 10.1002/hec. 4730020305; ^3^Teleman P, Stenzelius K, Iorizzo L, Jakobsson U. Validation of the Swedish short forms of the pelvic floor impact questionnaire (PFIQ-7), pelvic floor distress inventory (PFDI-20) and pelvic organ prolapse/urinary incontinence sexual questionnaire (PISQ-12). Acta Obstet Gynecol Scand. 2011;90(5):483-7.; ^4^Lundin A, Leijon O, Vaez M, Hallgren M, Torgen M. Predictive validity of the Work Ability Index and its individual items in the general population. Scand J Public Health. 2017:45(4):350-6.

An online questionnaire was developed in Swedish. Study data were collected and managed using REDCap electronic data capture tools hosted at Umeå University (51, 52). REDCap is a secure, web-based software platform designed to support data capture for research studies, providing 1) an intuitive interface for validated data capture; 2) audit trails for tracking data manipulation and export procedures; 3) automated export procedures for seamless data downloads to common statistical packages; and 4) procedures for data integration and interoperability with external sources (51, 52). The questionnaire employed for data collection in this study, comprising eight sections and a total of 130 items, was designed to require approximately 30 min for completion (Figure 1). The initial sections gathered demographic data, lifestyle choices, and reproductive health information. The latter sections employed five validated instruments assessing HRQoL, symptoms, and bother from the pelvic floor, sexual functioning, and work ability. More specifically, HRQoL was assessed using a combination of instruments that evaluate HRQoL, such as the RAND 36-Item Health Survey 1.0 (53–55) (included in this paper), as well as condition-specific HRQoL instruments (not reported here). The questionnaire underwent pilot testing with two women facing SPT-related health problems and eight healthcare professionals at Umeå University, resulting in minor adjustments to the language and programming of the questionnaire. Public involvement in the research project played a crucial role in both study design regarding the content of the questionnaire and assistance during social media outreach for data collection. Participants were required to confirm their eligibility and provide written informed consent before accessing the questionnaire. Access was revoked for those who did not meet these criteria. Data collection occurred from November 20, 2023, to March 15, 2024, with two reminders on the same online channels issued after 2–3 weeks and an additional 4 weeks, respectively.

#### 2.4.1 Outcome variable – HRQoL

The RAND 36-Item Health Survey 1.0 (RAND-36) (53–55) is one of the most widely utilized and validated patient-reported outcome measures assessing HRQoL globally. It consists of 36 items, selected from a broader set used in the RAND Medical Outcomes Study. It evaluates eight health concepts, as shown in Table 1, using multi-item scales, including physical functioning, role limitations due to physical and emotional health problems, social functioning, emotional well-being, energy/fatigue, pain, and general health perceptions. Additionally, it features a single item that assesses perceived changes in health over the past year. The scoring of RAND-36 involves a two-step process wherein a scoring key is initially applied to each item. Subsequently, these items are averaged to yield eight scale scores, each ranging from 0 to 100. Consequently, a higher score denotes a more favorable health status (53–55). Composite summary scores for physical and mental health (56) can be computed by aggregating their respective subscales into Physical Composite Summary (PCS) and Mental Composite Summary (MCS) scores, please see Table 1 and Supplementary Figure S1.

**Table 1:** Overview of variables included in linear regression model.

Continuous variable	Outcome/predictor	Description	Validated	Number of items	Scoring	Scales/groups
Age	Sociodemographic predictor	Age in years Self-reported data.	N/A	1 item	Derived from the participant's year of birth	Single scale variable
RAND-36	Outcome	RAND 36-Item Health Survey 1.0 assessing health-related quality of life on eight health concepts. Self-reported data.	English^a^ Swedish^b^	36 items	0 (worst health status)−100 (best health status) on all scales Scoring key according to RAND Health Care®^c^	8 subscales: Physical functioning* Role limitations due to physical problems* Role limitations due emotional health problems** Social functioning** Emotional well-being** Energy/fatigue** Pain* General health perception* Single item scale: Health change during last year 2 composite summary scales: Physical health (PCS)* Mental health (MCS)**
Symptom bother from SPT	Obstetric predictor	Visual analogue scale assessing symptom bother from SPT. Self-reported data.	Not validated for use on symptom bother	1 item	0 (no bother)−10 (extreme bother) on integer scale	Single scale variable
Total WAI-score	Occupational predictor	Work Ability Index (WAI) assessing level of work ability. Self-reported data.	English^d^ Swedish^e^^,^^f^	7 items	7 (poor work ability) – 49 points (excellent work ability) Scoring key^d^	Single score variable 4 groups: Poor (7−27 points) Moderate (28−36 points) Good (37−43 points) Excellent (44−49 points)

Categorical variable	Outcome/predictor	Description	Scales/groups
Degree of SPT	Obstetric predictor	Third-degree: partially torn anal sphincter muscle; Fourth-degree: entirely torn anal sphincter muscle including anorectal mucosa; When multiple SPT: highest degree of SPT included	Group 0 (reference): third-degree perineal laceration Group 1: fourth-degree perineal laceration
Education level	Sociodemographic predictor	6 educational groups collapsed into 2 groups: University level: university or college−less than 3 years; university or college−more than 3 years; Primary or secondary level: elementary school, public school, secondary school, or similar (9 years or less); 2-years of high school or vocational training; 3–4 years of high school; folk high school or similar	Group 0 (reference): university level Group 1: primary or secondary level
Employment status	Occupational predictor	10 different employment statuses collapsed into 2 mayor groups: Employed: employed; self-employed; Other: student; unemployed; sick cash benefit; homemaker; other	Group 0 (reference): employed Group 1: other
Employment type	Occupational predictor	Total percentage of main employment collapsed into 2 groups: Full-time workers: 100%; Part-time workers: less than 100%	Group 0 (reference): full-time Group 1: part-time
History of sick leave	Occupational predictor	> 7 consecutive days requiring sick leave certificate from physician; 4 groups collapsed into 2 groups: No sick leave: no sick leave; Sick leave: sick leave due to SPT; sick leave due to both SPT and other diagnosis; sick leave due to other diagnosis	Group 0 (reference): no sick leave Group 1: sick leave
Level of physical activity	Sociodemographic predictor	5-point Likert scale collapsed into 3 groups comparing current level of physical activity to before SPT: Same level: same Higher level: more; much more; Lower level: less; much less	Group 0 (reference): same level than before SPT Group 1: higher level than before SPT Group 2: lower level than before SPT
Parity	Obstetric predictor	Number of births collapsed into 2 groups: Primiparous: 1 birth Multiparous: > 1 birth	Group 0 (reference): primiparous Group 1: multiparous
Reconstructive surgery	Obstetric predictor	4 groups collapsed into 2 groups: Surgery: yes – once; yes - multiple times; No surgery: no surgery; on waiting list	Group 0 (reference): surgery Group 1: no surgery
Tobacco use	Sociodemographic predictor	Tabacco is defined as cigarettes, cigarillos, pipes, e-cigarettes, joints, or snuff. 3 groups collapsed into 2 groups: Tobacco use: daily use; sometimes; No tobacco use: no tobacco	Group 0 (reference): no tobacco use Group 1: tobacco use

a Hays R, Sherbourne C, Mazel R. The Rand 36-item Health Survey 1.0. Health Econ. (1993) 2(3):217–27. doi: 10.1002/hec.4730020305.

b Orwelius L, Nilsson M, Nilsson E, Wenemark M, Walfridsson U, Lundstrom M, et al. The Swedish RAND-36 Health Survey−reliability and responsiveness assessed in patient populations using Svensson's method for paired ordinal data. J Patient Rep Outcomes. (2017) 2(1):4. doi: 10.1186/s41687-018-0030-0.

c RAND Health Care. 36-Item Short Form Survey (SF-36) Scoring Instructions. (2025) https://www.rand.org/health-care/surveys_tools/mos/36-item-short-form/scoring.html [Accessed May 8, 2025].

d Tuomi K IJ. Work Ability Index. 2nd revised ed. Helsinki: Finnish Institute of Occupational Health. (1998).

e Torgén M. Experiences of WAI in a random sample of the Swedish working population. Int Congr Ser. (2005) 1280:328–32.

f Lundin A, Leijon O, Vaez M, Hallgren M, Torgen M. Predictive validity of the Work Ability Index and its individual items in the general population. Scand J Public Health. (2017). 45:350–6.

Based on previous Swedish findings (41), who presented Swedish reference data showing a mean average score of 66.9 (SD 23.5) for women in the domain “general health”, we hypothesized a decline of at least 7.0 (i.e., decline from 66.9 to 59.9 in mean score in general health representing a small to medium effect size) among women with long-term SPT-related health problems. To achieve an 80% power at a 5% significance level, a minimum of 178 participants were required. Accounting for a 10% dropout rate due to potentially missing data, we aimed to include approximately 197 participants in the final sample.

#### 2.4.2 Potential predictor variables

Clinically and theoretically relevant independent variables were identified based on literature reviews and clinical expertise within the research group. The set of variables encompassed sociodemographic, obstetric, and occupational information relevant to HRQoL, as shown in Table 1. Nine categorical variables were included; education level, tobacco use, parity, degree of SPT (Supplementary Figure S2), reconstructive surgery, employment status, employment type, history of sick leave (>7 consecutive days requiring sick leave certificate from physician) in adult life, and level of physical activity compared to before SPT. Age, symptom bother from SPT, and total Work Ability Index (WAI) score were continuous variables, and their properties are also presented in Table 1.

### 2.5 Data analysis

A data analysis plan was outlined prior to initiation of data analysis and discussed with a statistician. Descriptive statistics are expressed as numbers (n) and their associated percentages. A missing data analysis revealed randomness in the missing data and a non-monotone pattern with 1.1% of values missing in total (Supplementary Data S1). Hence, missing data was addressed by applying a fully conditional specification multiple imputation (Markov chain Monte Carlo method) with five imputations (57).

The RAND-36 was scored by following the official scoring instructions provided by RAND Health Care® (58), as shown in Supplementary Figure S1. Unanswered items were excluded from this calculation, so the scale scores reflect the average of only those items that the respondent completed (54). The PCS score for physical health (56) was calculated by summing the subscale scores for physical functioning, role limitations due to physical health problems, pain, and general health perceptions, and then dividing by four. Similarly, the MCS score for mental health was derived by adding the scores for energy/fatigue, social functioning, role limitations due to emotional health problems, and emotional well-being, and dividing by four (56). A one-sample *t*-test was performed to evaluate whether the mean score of PCS and MCS differed from the reference population's mean PCS of 73.625 (*SD* = 29.45) and mean MCS of 71.7 (*SD* = 27.15). Data on Women’s mean RAND-36 scores in Sweden published by Ohlsson-Nevo et al. (41) were used as the reference population. Further, the raw mean RAND-36 scores in our sample were standardized by calculating *T*-scores, with a mean of 50 and a standard deviation of 10, to visualize the difference between the mean RAND-36 scores in our sample and those in the general population in Sweden (41). Floor and ceiling effects of the RAND-36 were also examined.

A multiple linear regression analysis was performed on the imputed dataset to identify predictors of PCS and MCS after SPT (59). The two models were constructed in a single block, with theoretically and clinically *a priori* identified sociodemographic, obstetric, and occupational predictors (Table 1). The models display adjusted unstandardized coefficients (B) with 95% confidence intervals. After fitting the models, assumptions in linear regression, i.e., linearity, homoscedasticity, independent and normally distributed errors and multicollinearity, were confirmed. We could not detect any multicollinearity between predictors. Further, as outliers and influential cases in the dataset were identified, a sensitivity analysis was conducted to test the robustness of the findings, where cases with high residuals and high leverage were excluded and models were adjusted for the same predictors. A dropout analysis (Supplementary Table S1) was ultimately performed, comparing participants who provided only background data with those included in the linear regression models, applying independent-samples *t*-test on continuous data and the Chi-Squared test or Fisher's Exact Test on categorical data (59).

Data were analyzed using IBM SPSS Statistics version 29, with statistical significance determined at *p* < 0.05. The paper complies with the STROBE (Strengthening the Reporting of Observational Studies in Epidemiology) guidelines for cross-sectional studies (60) (Supplementary Table S2). The authors utilized Copilot® and Grammarly® for language editing of the manuscript, subsequently reviewing and refining the content language wise, and thus, assume full responsibility for the publication's accuracy.

### 2.6 Ethical considerations

The research project received ethical approval from the Swedish Ethical Review Authority on August 28, 2023 (Dnr: 2023-04018-01) and adheres to the Declaration of Helsinki (61). Participation was voluntary, allowing individuals to withdraw at any time without needing to provide a reason. Participants gave written informed consent digitally before accessing the questionnaire, which included options to skip intimate questions. The questionnaire was anonymous, with no email or IP addresses collected, and no incentives were provided. Furthermore, the funding organizations were not involved in any aspect of the study's design, data management, analysis, or the preparation and submission of the manuscript.

## 3 Results

### 3.1 Study sample and demographics of study population

**Figure 2:**
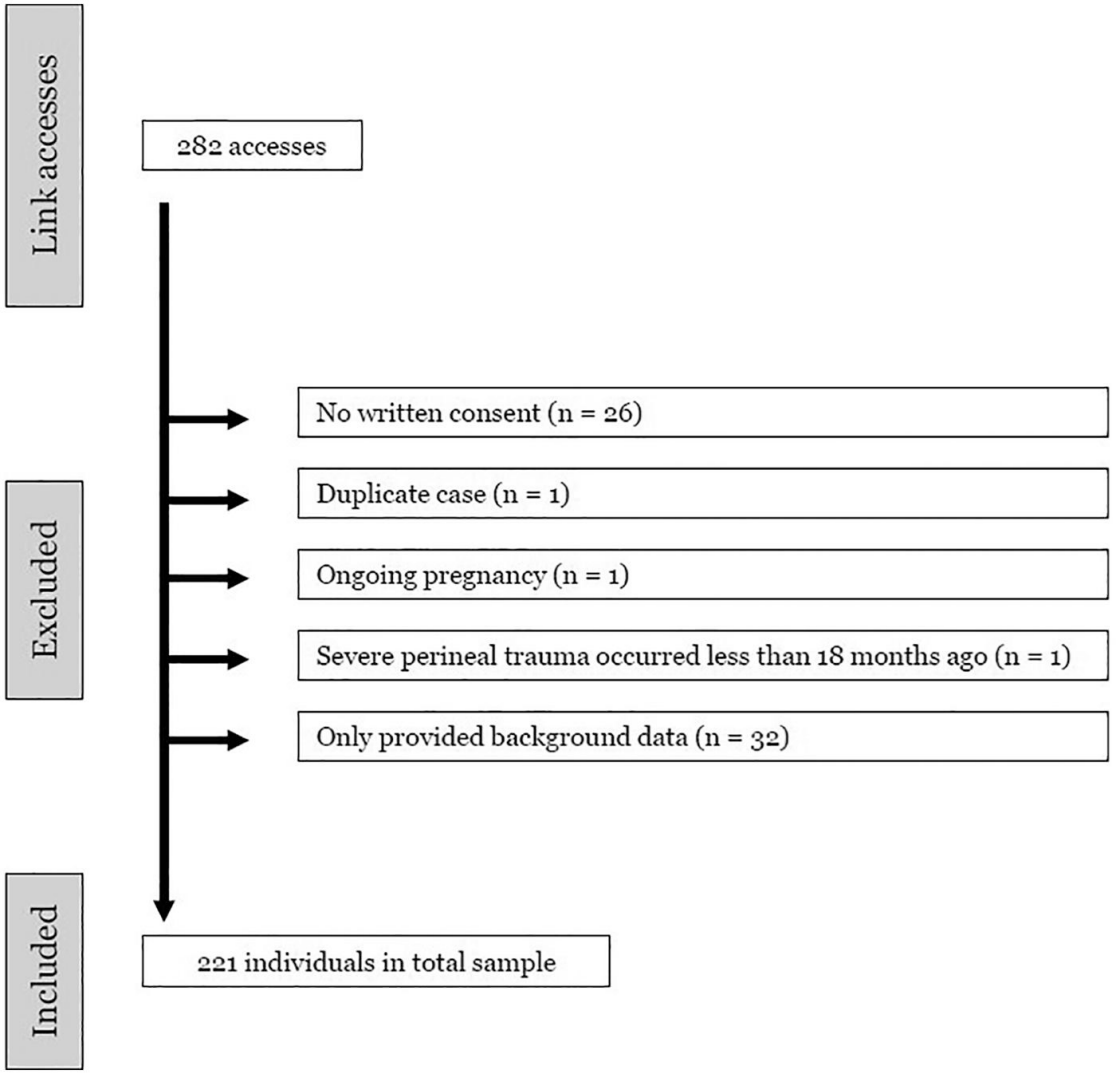
Inclusion and exclusion process.

The link to the questionnaire was accessed by a total of 282 individuals, with 255 individuals proceeding beyond the consent form. The inclusion procedure for this study is shown in Figure 2. The final sample consisted of 221 participants.

Table 2 provides an overview of the demographics of the total study population (*n* = 221), all identifying themselves as women. Most included women were born in Sweden (93.2%), had university-level education (81.9%), and cohabited with their partner (87.8%). Most women were employed (86.0%) or self-employed (4.1%), worked full-time (68.8%) and overall, the study population reported good work ability. Regarding obstetric properties, most women were multiparous (76.0%), and approximately one-third of the women sustained a fourth-degree perineal laceration (34.4%). On average, women had been living with SPT for 10.0 (*SD* 8.2; median 8.0, Q1−Q3: 4.0–13.0) years, and one third had pursued reconstructive surgery (30.3%). Based on self-reported symptom bother, the women were distributed evenly across three groups: mild (30.3%–31.7%), moderate (32.6%–33.9%), and severe symptom bother (34.8%–36.2%). The dropout analysis (Supplementary Table S1) revealed no significant differences in characteristics between women who dropped out and those who completed the questionnaire, except for the highest completed educational level. University-level education was overrepresented among included participants, and primary/secondary education was overrepresented among excluded participants.

**Table 2:** Demographics of study population (*n* = 221).

Variables	Raw data	Imputed data				
		Set 1	Set 2	Set 3	Set 4	Set 5
Sociodemographic variables						
Age in years						
Mean (SD; min-max)	40.0 (7.8; 24–71)	40.0 (7.7; 24–71)	40.0 (7.7; 24–71)	40.0 (7.8; 24–71)	40.0 (7.7; 24–71)	40.1 (7.9; 24–71)
Median (Q1-Q3; min-max)	38.0 (35–43; 24–71)	38.0 (35–43; 24–71)	38.0 (35–43; 24–71)	38.0 (35–43; 24–71)	38.0 (35–43; 24–71)	38.0 (35–43; 24–71)
Missing data, *n* (%)	1 (0.5)	0	0	0	0	0
Country of birth - Sweden, *n* (%)	206 (93.2)	209 (94.6)	209 (94.6)	207 (93.7)	210 (95.0)	209 (94.6)
Other country, *n* (%)	11 (5.0)	12 (5.4)	12 (5.4)	14 (6.3)	11 (5.0)	12 (5.4)
Missing data, *n* (%)	4 (1.8)	0	0	0	0	0
Highest completed educational level						
University, *n* (%)	181 (81.9)	183 (82.8)	184 (83.3)	182 (82.4)	182 (82.4)	183 (82.8)
Primary or secondary school, *n* (%)	37 (16.7)	38 (17.2)	37 (16.7)	39 (17.6)	39 (17.6)	38 (17.2)
Missing data, *n* (%)	3 (1.4)	0	0	0	0	0
Co-habiting with partner, *n* (%)	194 (87.8)					
Missing data, *n* (%)	0					
Tobacco user (cigarettes/cigarillos/pipes/e- cigarettes/joints/snuff), *n* (%)	30 (13.6)					
Missing data, *n* (%)	0					
Level of physical activity compared to before SPT^a^						
Same level of physical activity, *n* (%)	61 (27.6)					
Higher level of physical activity, *n* (%)	38 (17.2)					
Lower level of physical activity, *n* (%)	122 (55.2)					
Missing data, *n* (%)	0					
Obstetric variables						
Parity						
Primipara, *n* (%)	53 (24.0)					
Multipara, *n* (%)	168 (76.0)					
Missing data, *n* (%)	0					
Degree of SPT						
Third-degree, *n* (%)	145 (65.6)					
Fourth-degree, *n* (%)	76 (34.4)					
Missing data, *n* (%)	0					
Number of years living with SPT						
Mean (SD; min-max)	10.0 (8.2; 1–49)					
Median (Q1–Q3; min-max)	8.0 (4–13; 1–49)					
18 months to 5 years, *n* (%)	76 (34.4)					
6–10 years, *n* (%)	61 (27.6)					
More than 10 years, *n* (%)	84 (38.0)					
Missing data, *n* (%)	0					
Current diseases diagnosed by a physician						
Genitourinary disease, *n* (%)	8 (3.6)					
Digestive disease, *n* (%)	27 (12.2)					
Mental disorder, *n* (%)	54 (24.4)					
Missing data, *n* (%)	0					
Symptom bother from SPT^b^						
Mean (SD; min-max)	5.2 (2.8; 0–10)	5.2 (2.8; 0–10)	5.2 (2.8; 0–10)	5.2 (2.8; 0–10)	5.3 (2.8; 0–10)	5.2 (2.8; 0–10)
Median (Q1-Q3; min-max)	5.0 (3.0–7.0; 0–10)	5.0 (3.0–7.0; 0–10)	5.0 (3.0–7.0; 0–10)	5.0 (3.0–7.0; 0–10)	5.0 (3.0–7.0; 0–10)	5.0 (3.0–7.0; 0–10)
Mild symptom bother^c^ (1–3), *n* (%)	67 (30.3)	68 (30.8)	70 (31.7)	67 (30.3)	68 (30.8)	68 (30.8)
Moderate symptom bother^d^ (4–6), *n* (%)	72 (32.6)	74 (33.5)	74 (33.5)	75 (33.9)	73 (33.0)	75 (33.9)
Severe symptom bother^e^ (7–10), *n* (%)	77 (34.8)	79 (35.7)	77 (34.8)	79 (35.7)	80 (36.2)	78 (35.3)
Missing data, *n* (%)	5 (2.3)	0	0	0	0	0
Reconstructive surgery, *n* (%)	67 (30.3)	68 (30.8)	68 (30.8)	67 (30.3)	68 (30.8)	68 (30.8)
No reconstructive surgery, *n* (%)	153 (69.2)	153 (69.2)	153 (69.2)	154 (69.7)	153 (69.2)	153 (69.2)
Missing data, *n* (%)	1 (0.5)	0	0	0	0	0
Occupational variables						
Employment status						
Employed, *n* (%)	190 (86.0)					
Self-employed, *n* (%)	9 (4.1)					
Student, *n* (%)	11 (5.0)					
Other (unemployed: *n* = 2; sick cash benefit: *n* = 3; homemaker: *n* = 1; other: *n* = 2), *n* (%)	11 (5.0)					
Missing data, *n* (%)	0					
Employment rate						
Full-time (100%), *n* (%)	152 (68.8)	155 (70.1)	154 (69.7)	155 (70.1)	157 (71.0)	162 (73.3)
Part-time (75% or more), *n* (%)	40 (18.1)	50 (22.6)	43 (19.5)	52 (23.5)	49 (22.2)	43 (19.5)
Part-time (less than 75%), *n* (%)	13 (5.9)	16 (7.2)	24 (10.9)	14 (6.3)	15 (6.8)	16 (7.2)
Missing data, *n* (%)	16 (7.2)	0	0	0	0	0
History of sick leave in adult life^f^						
No sick leave, *n* (%)	118 (53.4)	118 (53.4)	119 (53.8)	118 (53.4)	119 (53.8)	118 (53.4)
Sick leave due to SPT, *n* (%)	29 (13.1)	30 (13.6)	29 (13.1)	29 (13.1)	29 (13.1)	29 (13.1)
Sick leave due to SPT and other diagnosis, *n* (%)	18 (8.1)	18 (8.1)	18 (8.1)	19 (8.6)	18 (8.1)	19 (8.6)
Sick leave due to other diagnosis, *n* (%)	55 (24.9)	55 (24.9)	55 (24.9)	55 (24.9)	55 (24.9)	55 (24.9)
Missing data, *n* (%)	1 (0.5)	0	0	0	0	0
WAI total score^g^						
Mean (SD; min-max)	38.9 (7.4; 13–49)	38.8 (7.3; 13–49)	38.8 (7.2; 13–49)	38.8 (7.5; 13–49)	38.6 (7.3; 13–49)	38.9 (7.7; 13–49)
Median (Q1-Q3; min-max)	41.0 (35.5–44.0; 13–49)	40.0 (35.0–44.0; 13–49)	40.0 (34.5–44.0; 13–49)	41.0 (35.0–44.3; 13–49)	40.0 (34.7–44.0; 13–49)	41.0 (35.5–44.7; 13–49)
Poor work ability (7–27 points), *n* (%)	18 (8.1)	22 (10.0)	30 (13.6)	30 (13.6)	25 (11.3)	20 (9.0)
Moderate work ability (28–36 points), *n* (%)	43 (19.5)	50 (22.6)	48 (21.7)	46 (20.8)	52 (23.5)	48 (21.7)
Good work ability (37–43 points), *n* (%)	81 (36.7)	87 (39.4)	85 (38.5)	84 (38.0)	82 (37.1)	82 (37.1)
Excellent work ability (44–49 points), *n* (%)	55 (24.9)	62 (28.1)	58 (26.2)	61 (27.6)	62 (28.1)	71 (32.1)
Missing data, *n* (%)	24 (10.9)	0	0	0	0	0

a SPT = severe perineal trauma.

b Symptom bother from SPT assessed on visual analogue scale (min-max: 0–10). 0 = no symptom bother from SPT; 10 = extreme symptom bother from SPT.

c Mild symptom bother from SPT rated as 0–3 on visual analogue scale.

d Moderate symptom bother from SPT rated as 4–6 on visual analogue scale.

e Severe symptom bother from SPT rated as 7–10 on visual analogue scale.

f Sick leave beyond one week requiring a sick leave certificate from a physician.

g Work Ability Index (WAI): min 7 points (poor work ability) - max 49 points (excellent work ability).

### 3.2 Health-related quality of life

Table 3 depicts the RAND-36 score distribution across the 8 subscales, the health change (single item), and the two composite summary scores for physical and mental health (PCS and MCS). Participants scored the lowest on the subscale energy/fatigue [mean 46.9, *SD* (Standard deviation) 20.3, *CI* (Confidence interval) 95% 44.2–49.6] and the highest on physical functioning (mean 82.8, *SD* 19.8, *CI* 95% 80.1–85.4). The single-item health change was rather stagnant at 54.5 (*SD* 21.6; *CI* 95% 51.6–57.4), suggesting improvement in overall health status during the past year. The mean PCS score was 70.7 (*SD* 22.1, *CI* 95% 67.7–73.6), while the mean MCS score was 63.2 (*SD* 21.4; *CI* 95% 60.4–66.0). The mean PCS for women in our sample (*M* = 70.7, *SD* = 22.1) was significantly lower than in the reference population (41) of women in Sweden (*M* = 73.63, *SD* = 29.45), *t* [*df* (degrees of freedom) 220] = −1.99, *p* = 0.047, Cohen's d = 0.13. Similarly, the mean MCS in our sample of women (*M* = 63.2, *SD* = 21.4) was significantly lower than in the reference population (41) of women in Sweden (*M* = 71.7, *SD* = 27.15), *t* (*df* 220) = −5.90, *p* < 0.001, Cohen's d = 0.40. Additionally, our sample reported worse-than-average RAND-36 scores compared to the reference population (visualized in *T*-scores) across all RAND-36 subscales and composite summary scores except for physical functioning and pain. The data was predominantly skewed, and the dimensions of role limitations due to physical health and due to emotional problems displayed floor effects. Further, physical functioning, role limitations due to physical health, role limitations due to emotional problems, social functioning, and pain showed ceiling effects.

**Table 3:** RAND-36^a^ score distribution in 8 subscales, health change and composite scores (*n* = 221).

Statistical measures	Physical functioning	Role limitations due to physical health	Role limitations due to emotional problems	Energy/fatigue	Emotional well-being	Social functioning	Pain	General health	Health change^b^	Physical composite score^c^	Mental composite score^d^
Raw scores											
Mean ^raw score^ (*SD*)^e^	82.8 (19.8)	67.8 (40.6)	65.4 (40.7)	46.9 (20.3)	67.5 (16.7)	73.0 (24.0)	74.4 (24.9)	57.8 (20.9)	54.5 (21.6)	70.7 (22.1)	63.2 (21.4)
*CI* 95%^f^	80.1–85.4	62.4–73.3	59.9–70.8	44.2–49.6	65.3–69.7	69.9–76.3	71.2–77.9	55.0–60.6	51.6–57.4	67.7–73.6	60.4–66.0
Median (*IQR*)^g^	90.0 (75.0–95.0)	100.0 (25.0–100.0)	100.0 (33.3–100.0)	45.0 (30.0–65.0)	68.0 (56.0–80.0)	75.0 (50.0–100.0)	80.0 (57.5–100.0)	60.0 (42.5–75.0)	50.0 (50.0–75.0)	77.5 (56.3–88.8)	66.9 (47.5–82.3)
Skewness	−1.543	−0.759	−0.606	−0.047	−0.596	−0.666	−0.678	−0.099	0.035	−0.783	−0.481
Kurtosis	1.953	−1.125	−1.300	−0.637	0.306	−0.250	−0.603	−0.688	0.240	−0.421	−0.859
Range	1–100	1–100	1–100	1–100	1–100	1–100	1–100	1–100	1–100	1–100	1–100
Floor (%)^h^	0.5	19.9	20.4	0.5	0.5	0.9	0.9	0.5	2.7	0.5	0.5
Ceiling (%)^i^	23.1	53.8	52.0	0.9	0.9	27.6	30.3	2.3	6.8	1.4	0.5
Missing data *n* (%)	0	0	0	0	0	0	0	0	3 (1.4)	0	0
Normal distribution	No	No	No	Yes	No	No	No	Yes	Yes	No	No
*T*-test (df)^j^										−1.99 (220)	−5.90 (220)
*p*-value^k^										**0.047**	**<0.001**
T-scores											
Mean ^t−score^ (*SD*)^l^	50.5 (7.6)	48.7 (9.9)	48.0 (10.2)	45.1 (8.5)	46.7 (8.4)	46.9 (9.5)	50.5 (9.1)	46.1 (8.9)	N/A	49.0 (7.5)	46.9 (7.9)

a RAND 36-Item Health Survey 1.0.

b Single item question assessing change in health during the last year.

c Average of subscales physical functioning, role limitations due to physical health, pain, and general health.

d Average of subscales energy/fatigue, social functioning, role limitations due to emotional problems, and emotional well-being.

e Standard deviation (*SD*). Higher mean scores reflect better health-related quality of life.

f 95% confidence interval (*CI*).

g Inter-quartile range (*IQR*).

h Proportion of participants with lowest subscale score.

i Proportion of participants with highest subscale score.

j One-sample *t*-test comparing mean raw PCS and MCS score to mean PCS and MCS of reference population of Swedish women (PCS = 73.63; MCS = 71.70) calculated from Table 4 in Ohlsson-Nevo E, Hiyoshi A, Norén P, Möller M, Karlsson J. The Swedish RAND-36: psychometric characteristics and reference data from the Mid-Swed Health Survey. J Patient-Rep Outcomes. 2021;5(1):66.

k Significance level set at *p* < 0.005. Bold values indicate statistically significant differences from reference population.

l T-scores are standardized raw scores with a mean of 50 and a standard deviation of 10. Reference population of Swedish women found in Table 4 in Ohlsson-Nevo et al. (2021). Calculated with the following formula where X is the scale raw score: $T=50+10\left(\frac{X-Mean^{reference}}{SD^{reference}}\right)$

### 3.3 Predictors of RAND-36 physical and mental health

#### 3.3.1 Physical health (PCS)

All five imputed multiple regression models with PCS as outcome were statistically significant (*p* < 0.001), explaining 63.0%–65.8% of the variance in PCS (see Table 4). The pooled multiple regression model depicted symptom bother from SPT, total WAI-score, education level, and level of physical activity as significant predictors of PCS. For every one unit in-crease in symptom bother from SPT, PCS decreased on average by 2.978 units. Further, for every one unit increase in total WAI-score (i.e., improved work ability), PCS increased on average by 1.167 units. Women with primary or secondary level education had, on average, 6.117 units lower PCS than women with university level education. Finally, women with lower level of physical activity than before SPT had, on average, 9.162 units lower PCS than women who remained physically active at the same level as before the SPT. These interpretations are accurate only if all other factors are held constant. The linear model of PCS remained stable after a sensitivity analysis excluding two influential cases, see Table 5.

**Table 4:** Pooled results of linear regression on predictors of RAND-36^a^ physical and mental health, *n* = 221.

Predictors	Reference group	*B*	S.E.	*t*	*p* ^b^	Lower 95% *CI*	Upper 95% *CI*
PCS^c^							
Symptom bother^d^	N/A	−2.978	0.395	−7.532	<0.001	−3.754	−2.203
Total WAI-score^e^	N/A	1.167	0.163	7.180	<0.001	0.845	1.489
Primary- or secondary-level of education	University-level education	−6.177	2.740	−2.254	0.025	−11.571	−0.783
Lower level of physical activity than before SPT^f^	Same level	−9.162	2.325	−3.940	<0.001	−13.721	−4.603
Constant	N/A	42.747	9.708	4.403	<0.001	23.519	61.976
MCS^g^							
Age^h^	N/A	0.361	0.158	2.289	0.023	0.050	0.671
Symptom bother^d^	N/A	−1.171	0.431	−2.718	0.007	−2.015	−0.326
Total WAI-score^e^	N/A	1.641	0.172	9.542	<0.001	1.302	1.980
History of sick leave in adult life	No history of sick leave	−5.405	2.636	−2.051	0.042	−10.610	−0.200
Constant	N/A	−2.800	10.133	−0.276	0.782	−22.744	17.144

a RAND 36-Item Health Survey 1.0.

b Significance level set at *p* < 0.005.

c Physical health = physical composite summary in RAND-36. Original data and imputed data models also adjusted for age, tobacco use, parity, degree of SPT, reconstructive surgery, employment status, employment type and history of sick leave in adult life. R^2^_original_ 0.658, R^2^_imputed 1_ 0.637, R^2^_imputed 2_ 0.654, R^2^_imputed 3_ 0.646, R^2^_imputed 4_ 0.638, R^2^_imputed 5_ 0.630. *F*-tests on original data and imputed data models are all significant at *p* < 0.001.

d Symptom bother assessed on visual analogue scale; range 0 (no bother) - 10 (extreme bother).

e Total WAI-score: Work Ability Index score; range 7 (poor) - 49 points (excellent) work ability.

f SPT = severe perineal trauma.

g Mental health = mental composite summary in RAND-36. Original data and imputed data models also adjusted for educational level, tobacco use, level of physical activity compared to before SPT, parity, degree of SPT, reconstructive surgery, employment status, and employment type. R^2^_original_ 0.588, R^2^_imputed 1_ 0.557, R^2^_imputed 2_ 0.558, R^2^_imputed 3_ 0.545, R^2^_imputed 4_ 0.544, R^2^_imputed 5_ 0.546. *F*-tests on original data and imputed data models are all significant at *p* < 0.001.

h Age: women between 24 and 71 years of age.

**Table 5:** Sensitivity analysis−pooled results of linear regression on predictors of RAND-36^a^ physical and mental health.

Predictors	Reference group	*b*	S.E.	*t*	*P* ^b^	Lower 95% *CI*	Upper 95% *CI*
PCS^c^ (*n* = 219)							
Symptom bother^d^	N/A	−2.883	0.385	−7.493	<0.001	−3.638	−2.129
Total WAI-score^e^	N/A	1.258	0.163	7.718	<0.001	0.935	1.582
Primary- or secondary-level of education	University-level education	−5.947	2.762	−2.153	0.033	−11.399	−0.496
Lower level of physical activity than before SPT^f^	Same level	−7.888	2.336	−3.377	<0.001	−12.470	−3.306
Constant	N/A	33.770	9.606	3.515	<0.001	14.835	52.705
MCS^g^ (*n* = 220)							
Age^h^	N/A	0.420	0.153	2.744	0.006	0.119	0.720
Symptom bother^d^	N/A	−1.112	0.430	−2.583	0.010	−1.956	−0.267
Total WAI-score^e^	N/A	1.676	0.181	9.268	<0.001	1.316	2.037
History of sick leave in adult life	No history of sick leave	−4.967	2.699	−1.841	0.069	−10.332	0.397
Constant	N/A	−7.634	10.474	−0.729	0.467	−28.337	13.068

a RAND 36-Item Health Survey 1.0.

b Significance level set at *p* < 0.005.

c Physical health = physical composite summary in RAND-36. Sensitivity analysis excluding case 108 and 148, stable model. Original data and imputed data models also adjusted for age, tobacco use, parity, degree of SPT, reconstructive surgery, employment status, employment type and history of sick leave in adult life. R^2^_original_ 0.647, R^2^_imputed 1_ 0.627, R^2^_imputed 2_ 0.647, R^2^_imputed 3_ 0.629, R^2^_imputed 4_ 0.62, R^2^_imputed 5_ 0.624. *F*-tests on original data and imputed data models are all significant at *p* < 0.001.

d Symptom bother assessed on visual analogue scale; range 0 (no bother) - 10 (extreme bother).

e Total WAI-score: Work Ability Index score; range 7 (poor) - 49 points (excellent) work ability.

f SPT = severe perineal trauma.

g Mental health = mental composite summary in RAND-36. Sensitivity analysis excluding case 148, history of sick leave in adult life did not remain a significant predictor of MCS. Original data and imputed data models also adjusted for educational level, tobacco use, level of physical activity compared to before SPT, parity, degree of SPT, reconstructive surgery, employment status, and employment type. R^2^_original_ 0.588, R^2^_imputed 1_ 0.565, R^2^_imputed 2_ 0.571, R^2^_imputed 3_ 0.538, R^2^_imputed 4_ 0.543, R^2^_imputed 5_ 0.564. *F*-tests on original data and imputed data models are all significant at *p* < 0.001.

h Age: women between 24 and 71 years of age.

#### 3.3.2 Mental health (MCS)

All five imputed multiple regression models with MCS as outcome were statistically significant (*p* < 0.001), explaining 54.4%–58.8% of the variance in MCS, see Table 4. The pooled multiple regression model depicted age, symptom bother from SPT, total WAI score, and history of sick leave as significant predictors of MCS. However, in a sensitivity analysis excluding one influential case (Table 5), history of sick leave did not remain a significant predictor of MCS. For every one-year increase in age, MCS increased on average by 0.361 units. For every one unit increase in symptom bother, MCS decreased, on average, by 1.171 units. Further, for every one unit increase in total WAI-score (work ability), MCS increased on average by 1.641 units. These interpretations are accurate only if all other factors are held constant.

## 4 Discussion

This is the first study to provide a comprehensive analysis of the HRQoL and associated predictors among a population of women living with SPT in Sweden. Notably, our study population exhibited worse-than-average RAND-36 scores across most dimensions (apart from physical functioning and pain) as well as significantly lower physical and mental health compared to normative data for women in Sweden (41). This indicates that the consequences of SPT may negatively impact physical and mental health aspects for a substantial number of women with SPT. The average PCS score among the study participants was 70.7 (out of 100), in contrast to the mean MCS score of 63.2 (out of 100). Women with SPT exhibited the lowest scores regarding energy/fatigue, while achieving the highest scores on the physical functioning subscale. The assessment of health change over the past year remained relatively static, suggesting a slight trend towards improvement in overall health status. Factors significantly predicting physical health included the extent of symptom bother from SPT, increasing perceived work ability, educational attainment, and level of physical activity. Mental health was significantly predicted by increasing age, the extent of symptom bother from SPT, and increasing perceived work ability. If sick leave has an association with mental health remains unclear.

Notably, our sample scored lower (i.e., reported lower HRQoL) on all RAND-36 subscales than women with SPT participating in a UK study comparing RAND-36 scores based on severity of SPT (25). However, this study and other research on HRQoL among women with SPT using RAND-36 struggle with small sample sizes (16, 25, 36) and large drop-out rates (16, 25), making comparisons challenging and emphasizing the significant contribution of our study to the research field. Although our findings pertaining to physical health exhibited considerable robustness, sick leave no longer emerged as a statistically significant predictor of mental health after the sensitivity analyses. Consequently, this study is unable to draw definitive conclusions regarding the association between sick leave and mental health outcomes. Considering that individuals on sick leave in Sweden in general report low HRQoL (41), further research is warranted to elucidate the potential role of sick leave in influencing mental health after SPT.

Women with SPT in our study reported significantly lower mental health than the general population in Sweden (41). Further, they scored lowest on energy and fatigue, a finding that resonates with other research on HRQoL after SPT (16, 25, 36), suggesting that women experiencing SPT often endure fatigue and decreased overall vitality. Considering that our sample reported comparable physical functioning to the general population in Sweden (41), this suggests that women with SPT may receive adequate physical recovery measures from the Swedish healthcare system, such as reconstructive surgery, urotherapeutical treatment, or pelvic floor rehabilitation. However, the presence of reconstructive surgery did not demonstrate any significant association with mental or physical health outcomes in our sample. This raises the question of whether the surgical techniques currently employed are effective in mitigating symptoms following SPT. Consequently, further research is warranted to evaluate and optimize approaches to secondary repair. Nevertheless, low mental health scores in contrast to high scores in physical functioning point towards a nuanced health status characterized by functional capacity that does not necessarily align with overall well-being – a health status requiring more resources targeting mental health outcomes.

Interestingly, neither reconstructive surgery nor the degree of SPT was associated with mental or physical health in our sample, whereas self-assessed symptom bother from SPT and work ability both demonstrated significant predictive value for these domains. In this context, personal experiences of how SPT symptoms impact daily life or working life might play a more prominent role than the sole existence of symptoms and may have a significant bearing on overall well-being after SPT. Research has linked SPT to psychological consequences like depression, anxiety (7), shame, guilt, isolation (2, 5), and negative impact on relationships, such as to their partner (2, 5), children (5), and the broader social networks in daily life (2, 5) and at work (19). Our findings demonstrated that increased symptom bother from SPT was associated with poorer physical and mental health, despite high physical functioning scores−highlighting the significant role of personal experiences and mental health in overall well-being and HRQoL. Another shared predictive factor of physical and mental health was self-assessed work ability, indicating that those with higher work ability are likely to report better HRQoL. This further underscores the multifaceted aspects of living with SPT and accentuates the need to focus on the psychological impact of symptoms and coping as a critical component of care and rehabilitation for these women. Recovery could be facilitated by co-creation in care and rehabilitation with women with SPT in research, by incorporating a psychologist in the recovery process, and by enabling rehabilitation and return to work in collaboration with employers (8, 62).

Further, our sample assessed their health change over the past year as relatively static, suggesting minimal improvement in overall health status. Women in our sample had an average duration of living with SPT of approximately ten years. Research on postpartum HRQoL among women in general (9) indicates a decline in postpartum HRQoL over the first three years. Our sample reported lower RAND-36 subscale scores in all domains except emotional well-being than postpartum women in general during that timeframe (9). Combined with scoring significantly lower on overall physical and mental health than the general population in Sweden (41), our results might indicate a potential development of a chronic condition that may lead to cumulative effects on health and well-being. Especially as postpartum HRQoL appears to decline over time, and SPT is identified as a significant risk factor for decreased postpartum QoL (9). In this context, prevention of SPT represents a critical priority, both in clinic and research (63).

Moreover, a lower physical activity level after SPT was a significant predictor of reporting lower physical health in our sample. A large body of evidence exists on the positive effects of physical activity on mortality rates and QoL, especially for individuals with mental health conditions (64). However, the level of physical activity decreases for both women and men postpartum (65). In general, research has linked postpartum physical activity to reduced symptoms of depression (66), anxiety (67), and fatigue (68), as well as improved maternal sleep quality (69), well-being (66), and mental health (70). Nevertheless, evidence on physical activity among women with SPT is almost nonexistent, as women often report an impaired ability to exercise (71); this is also supported by our findings, in which 55.2% reported a lower level of physical activity than before SPT. Further, there is very limited evidence on how physical therapy and pelvic floor muscle training (72) might affect pelvic floor functioning and the ability to exercise after SPT. This suggests an urgent need to further investigate and facilitate the resumption of physical activity as a potential therapeutic avenue for women with SPT. In this context, rehabilitation programs should not only focus on pelvic floor training but also on the ability to pursue daily physical activity as part of treatment. Potentially, group-based physical activity (73) among women with SPT may increase postpartum physical activity levels and entail secondary positive outcomes, such as reduced social isolation. Hence, the benefit could be multidimensional as physical activity has also been shown to improve fatigue (68), which our sample of women scored lowest on.

Although the normative population (41) exhibit a higher mean age than the present study, participants in our sample reported lower HRQoL. This finding is noteworthy, as it underscores that many individuals in the study, despite having numerous years of potential active life ahead, might already be confronted with substantial health-related limitations. However, in our study, age was a significant predictor of mental health, where HRQoL scores increased with advancing age, contrasting with other research showing worse mental health in older adults (41). The increase in mental health scores may reflect greater resilience or the ability to cope developed over time as priorities and demands in daily life and working life shifts. This finding underscores the potential necessity to design age-appropriate, individualized interventions that cater to the specific needs and nuances of mental health in different generations of women living with SPT. However, further research is essential to elucidate the underlying dynamics behind these age-related differences in mental health and whether age as a predictor of mental health is isolated to our specific sample.

### 4.1 Strengths and limitations

A strength of the study is that we achieved a comparatively robust sample size despite the potentially sensitive nature of the topic. Moreover, our sample encompassed a spectrum of women reporting no symptom bother after SPT to those experiencing severe symptom bother, strengthening external validity. The use of a widely accepted and thoroughly validated instrument for measuring HRQoL (53–55) further strengthened the construct validity and reliability of this study. The standardization of scores presented as T-scores (74) facilitated comparison to the normative population, further enhancing the external validity of our findings.

However, there are sources of bias that need to be discussed. Our sample was predominantly composed of Swedish-born women with university-level education who cohabited with partners and were engaged in full-time employment, suggesting a considerable degree of socioeconomic stability in their lives, which may have introduced potential selection bias to the findings. Empirical evidence suggests that the presence of a supportive partner and robust social networks significantly contribute to individual well-being among middle-aged women (75). Therefore, research should expand beyond our cohort to include a more diverse range of participants, encompassing various ethnicities, educational backgrounds, and socioeconomic classes, which could facilitate a more in-depth understanding of HRQoL in women with SPT, especially in potentially vulnerable subgroups. Another potential selection bias was that approximately 30% of the women reported fourth-degree perineal laceration, contrasting with official Swedish reports (14, 76) that report an annual incidence of fourth-degree perineal laceration of 5%–10%, but the degree of perineal laceration was not associated with physical or mental health. The included data relies solely on patient-reported outcome measures (PROMs) collected via digital platforms. While PROMs provide valuable insights into subjective experiences, they are prone to information bias, including social desirability and recall bias (77), which may affect reliability. Moreover, the use of social media and patient organizations as recruitment channels may have disproportionately attracted individuals with particularly salient experiences, introducing information overload (78) and impacting the validity of results. To reduce the risk of information bias and strengthen the study's validity and reliability, validated instruments were incorporated into the questionnaire whenever feasible, except for background items. However, future research could benefit from incorporating objective health measures, derived from registers or medical records, alongside PROMs to improve the validity and reliability of results. The wide confidence intervals for some categorical predictors suggest caution in interpreting their associations with the outcome, highlighting the need for more precise measurement tools to improve reliability. Notably, significant ceiling effects were observed in the RAND-36 role limitation subscales, which limited the detection of variability among high-scoring participants−a limitation also reported in previous studies (41, 79). However, the use of composite summary scores in the regression model mitigated this issue, as they did not exhibit floor or ceiling effects. Floor and ceiling effects underscore the potential value of alternative scoring methods for RAND-36, such as item response theory (74), and the use of condition-specific QoL instruments to better capture HRQoL following SPT.

## 5 Conclusions

This study offers the first comprehensive assessment of HRQoL in women with SPT. Women with SPT reported significantly lower HRQoL compared to the general female population in Sweden, particularly in mental health domains. The results highlight the intricate interplay between physical symptoms and psychological well-being, underscoring the need for comprehensive care approaches. Personal experiences and perceived impact on daily life may be more influential than clinical aspects alone. The identification of vulnerable subgroups underscores the need for tailored multidisciplinary care strategies that address both physical and psychological recovery. Future research should further explore socioeconomic and occupational factors to inform equitable and effective health outcomes. Barriers and facilitators, especially the role of sick leave, influencing HRQoL in this population, need further investigation to enhance HRQoL and support the reintegration of women with SPT into their social and professional spheres.

## Data Availability

The datasets presented in this article are not readily available because Swedish law prioritizes protection of confidentiality of participants. Requests to access the datasets should be directed to Katharina Tjernström, katharina.tjernstrom@umu.se.

## References

[1] Socialstyrelsen [The National Board of Social Affairs and Health]. Internationell statistisk klassifikation av sjukdomarch och relaterade hälsoproblem, systematisk förteckning, Svensk version 2025, Del 2 (3) H–P [International Statistical Classification of Diseases and Related Health Problems, Swedish version 2025, Part 2 (3) H–P]. (2025). Available online at: https://www.socialstyrelsen.se/statistik-och-data/klassifikationer-och-koder/icd-10/ (Accessed December 30, 2025)

[2] Darmody E Bradshaw C Atkinson S. Women’s experience of obstetric anal sphincter injury following childbirth: an integrated review. Midwifery. (2020) 91:102820. 10.1016/j.midw.2020.102820 32861872

[3] LaCross A Groff M Smaldone A. Obstetric anal sphincter injury and anal incontinence following vaginal birth: a systematic review and meta-analysis. J Midwifery Womens Health. (2015) 60(1):37–47. 10.1111/jmwh.12283 25712278

[4] Samarasekera DN Bekhit MT Wright Y Lowndes RH Stanley KP Preston JP Long-term anal continence and quality of life following postpartum anal sphincter injury. Colorectal Dis. (2008) 10(8):793–9. 10.1111/j.1463-1318.2007.01445.x 18266886

[5] d’Almeida I. Women’s experiences following obstetric anal sphincter injury. J Pelvic Obstet Gynaecol Physiother. (2020) 127:39–50. Available online at: https://thepogp.co.uk/journal/10/pogp_journal_issue_127_autumn_2020/15/

[6] Andreucci CB Bussadori JC Pacagnella RC Chou D Filippi V Say L Sexual life and dysfunction after maternal morbidity: a systematic review. BMC Pregnancy Childbirth. (2015) 15:307. 10.1186/s12884-015-0742-6 26596506 PMC4657322

[7] Keighley MR Perston Y Bradshaw E Hayes J Keighley DM Webb S. The social, psychological, emotional morbidity and adjustment techniques for women with anal incontinence following obstetric anal sphincter injury: use of a word picture to identify a hidden syndrome. BMC Pregnancy Childbirth. (2016) 16(1):275. 10.1186/s12884-016-1065-y 27654450 PMC5031357

[8] Crookall R Fowler G Wood C Slade P. A systematic mixed studies review of women’s experiences of perineal trauma sustained during childbirth. J Adv Nurs. (2018) 74(9):2038–52. 10.1111/jan.13724 29791012

[9] Martínez-Galiano JM Hernández-Martínez A Rodríguez-Almagro J Delgado-Rodríguez M. Quality of life of women after giving birth: associated factors related with the birth process. J Clin Med. (2019) 8(3):324. 10.3390/jcm8030324 30866580 PMC6462924

[10] Gyhagen M Ellström Engh M Husslein H Koelbl H Nilsson IEK Schulz J Temporal trends in obstetric anal sphincter injury from the first vaginal delivery in Austria, Canada, Norway, and Sweden. Acta Obstet Gynecol Scand. (2021) 100(11):1969–76. 10.1111/aogs.14244 34435349

[11] Blondel B Alexander S Bjarnadóttir RI Gissler M Langhoff-Roos J Novak-Antolič Ž Variations in rates of severe perineal tears and episiotomies in 20 European countries: a study based on routine national data in euro-peristat project. Acta Obstet Gynecol Scand. (2016) 95(7):746–54. 10.1111/aogs.12894 26958827

[12] Laine K Fodstad K Räisänen S. Obstetric anal sphincter injuries in spontaneous vaginal births in nulliparous pregnant individuals: a 21-year cohort study based on real-world data. Am J Obstet Gynecol. (2025) 233(5):448.e1–448.e10. 10.1016/j.ajog.2025.06.014 40513930

[13] Socialstyrelsen [The National Board of Social Affairs and Health]. Statistikdatabas för graviditeter, förlossningar och nyfödda [Statistics database for pregnancies, births and newborns]. (2025). Available online at: https://sdb.socialstyrelsen.se/if_mfr_004/val.aspx (Accessed December 30, 2025)

[14] Pihl S. Bristning vid förlossning grad 3–4. Årsrapport från GynOp-registret avseende operationer utförda år 2024 [Grade 3–4 rupture of membranes. Annual report from the GynOp register for surgeries performed in 2024]. (2025). Available online at: https://www.gynop.se/wp-content/uploads/2025/04/Arsrapport-bristning-grad-3-4-atgard-utford-ar-2024.pdf (Accessed December 30, 2025)

[15] Ustjanauskas AE Malcarne VL. Health-related quality of life. In: Gulliver SB Cohen LM, editors. The Wiley Encyclopedia of Health Psychology, Vol 4. Chichester: John Wiley & Sons, Ltd (2020). p. 149–54. 10.1002/9781119057840.ch198

[16] Tilak M Mann GK Gong M Koenig NA Lee T Geoffrion R. Pelvic floor healing milestones after obstetric anal sphincter injury: a prospective case control feasibility study. Int Urogynecology J. (2023) 34(2):553–61. 10.1007/s00192-022-05348-6 PMC9469830 36098790

[17] Tjernström K Lindberg I Wiklund M Persson M. Overlooked by the obstetric gaze—how women with persistent health problems due to severe perineal trauma experience encounters with healthcare services: a qualitative study. BMC Health Serv Res. (2024) 24(1):610. 10.1186/s12913-024-11037-5 38724992 PMC11084138

[18] Murray C George M Davis J Edvardsson K. Women’s choices and preferences for subsequent mode of birth following an obstetric anal sphincter injury (OASI): a scoping review. Midwifery. (2025) 150:104588. 10.1016/j.midw.2025.104588 40934712

[19] Tjernström K Lindberg I Wiklund M Persson M. Negotiating the ambiguity of an (in)authentic working life: a grounded theory study into severe perineal trauma. BMC Womens Health. (2023) 23(1):47. 10.1186/s12905-023-02191-9 36750837 PMC9902817

[20] Rebmann E Hamel JF Helbert C Lemasson F Legendre G Venara A. Anal incontinence after obstetrical anal sphincter injury significantly impacts quality of life for women: a cohort study. Langenbecks Arch Surg. (2024) 409(1):67. 10.1007/s00423-024-03257-4 38368278

[21] Tucker J Clifton V Wilson A. Teetering near the edge; women’s experiences of anal incontinence following obstetric anal sphincter injury: an interpretive phenomenological research study. Aust N Z J Obstet Gynaecol. (2014) 54(4):377–81. 10.1111/ajo.12230 25117190

[22] Cornelisse S Arendsen LP van Kuijk SM Kluivers KB van Dillen J Weemhoff M. Obstetric anal sphincter injury: a follow-up questionnaire study on longer-term outcomes. Int Urogynecol J. (2016) 27(10):1591–6. 10.1007/s00192-016-3017-5 27085544

[23] Tejedor P Bodega-Quiroga I Plaza J Ortega López M Gutierrez C García Olmo D Quality of life and 3D-EUS assessment for anal incontinence after childbirth. Rev Esp Enferm Dig. (2019) 111(6):453–9. 10.17235/reed.2019.6040/2018 31021166

[24] Jangö H Langhoff-Roos J Rosthøj S Sakse A. Wexner score and quality of life in women with obstetric anal sphincter injury. Int Urogynecology J. (2020) 31(6):1115–21. 10.1007/s00192-019-04134-1 31792591

[25] Ramage L Yen C Qiu S Simillis C Kontovounisios C Tekkis P Functional and quality of life outcomes following obstetric anal sphincter injury (OASI): does the grade of injury affect outcomes? Int Urogynecology J. (2017) 28(11):1709–17. 10.1007/s00192-017-3334-3 PMC5655560 28523401

[26] Desseauve D Proust S Carlier-Guerin C Rutten C Pierre F Fritel X. Evaluation of long-term pelvic floor symptoms after an obstetric anal sphincter injury (OASI) at least one year after delivery: a retrospective cohort study of 159 cases. Gynecol Obstet Fertil. (2016) 44(7–8):385–90. 10.1016/j.gyobfe.2016.05.007 27451064

[27] Sartore A Scalia MS Mangino FP Savastano G Magni E Ricci G. Pelvic floor function after third- and fourth-degree perineal lacerations: a case-control study on quality of life. BMC Womens Health. (2024) 24(1):12. 10.1186/s12905-023-02739-9 38172805 PMC10765914

[28] Tin RYT Schulz J Gunn B Flood C Rosychuk RJ. The prevalence of anal incontinence in post-partum women following obstetrical anal sphincter injury. Int Urogynecol J. (2010) 21(8):927–32. 10.1007/s00192-010-1134-0 20422153

[29] Roos AM Thakar R Sultan AH. Outcome of primary repair of obstetric anal sphincter injuries (OASIS): does the grade of tear matter? Ultrasound Obstet Gynecol. (2010) 36(3):368–74. 10.1002/uog.7512 20069661

[30] Schütze S Hohlfeld B Friedl TWP Otto S Kraft K Hancke K Fishing for (in)continence: long-term follow-up of women with OASIS−still a taboo. Arch Gynecol Obstet. (2021) 303(4):987–97. 10.1007/s00404-020-05878-8 33258994 PMC7985110

[31] Webb SS Yates D Manresa M Parsons M MacArthur C Ismail KMK. Impact of subsequent birth and delivery mode for women with previous OASIS: systematic review and meta-analysis. Int Urogynecology J. (2017) 28(4):507–14. 10.1007/s00192-016-3226-y 28025682

[32] Fradet-Menard C Deparis J Gachon B Sichitiu J Pierre F Fritel X Obstetrical anal sphincter injuries and symptoms after subsequent deliveries: a 60 patient study. Eur J Obstet Gynecol Reprod Biol. (2018) 226:40–6. 10.1016/j.ejogrb.2018.05.007 29804027

[33] Nilsson IEK Åkervall S Molin M Milsom I Gyhagen M. Symptoms of fecal incontinence two decades after no, one, or two obstetrical anal sphincter injuries. Am J Obstet Gynecol. (2021) 224(3):276.e1–276.e23. 10.1016/j.ajog.2020.08.051 32835724

[34] Webb SS Sitch A MacArthur C. The impact of mode of subsequent birth after obstetric anal sphincter injury on bowel function and related quality of life: a cohort study. Int Urogynecology J. (2020) 31(11):2237–45. 10.1007/s00192-020-04234-3 PMC7561530 32095959

[35] Barbosa M Glavind-Kristensen M Christensen P. Early secondary repair of obstetric anal sphincter injury: postoperative complications, long-term functional outcomes, and impact on quality of life. Tech Coloproctol. (2020) 24(3):221–9. 10.1007/s10151-019-02146-z 32020351

[36] Visscher AP Lam TJ Hart N Felt-Bersma RJF. Fecal incontinence, sexual complaints, and anorectal function after third-degree obstetric anal sphincter injury (OASI): 5-year follow-up. Int Urogynecol J. (2014) 25(5):607–13. 10.1007/s00192-013-2238-0 24196652

[37] Maldonado PA McIntire D Corton MM. Long-Term outcomes after overlapping sphincteroplasty for cloacal-like deformities. Female Pelvic Med Reconstr Surg. (2019) 25(4):271–8. 10.1097/SPV.0000000000000543 29324570

[38] Oakley SH Ghodsi VC Crisp CC Estanol MV Westermann LB Novicki KM Impact of pelvic floor physical therapy on quality of life and function after obstetric anal sphincter injury: a randomized controlled trial. Female Pelvic Med Reconstr Surg. (2016) 22(4):205–13. 10.1097/SPV.0000000000000255 26829343

[39] Arcieri M Battello G Graziano A Alfarè Lovo M Restaino S D’Antonio F The outcome of early perineal rehabilitation in obstetric anal sphincter injuries: a single-center experience. Arch Gynecol Obstet. (2025) 311(6):1711–9. 10.1007/s00404-024-07906-3 39869199 PMC12055880

[40] Tjernström K. Beyond the “obstetric gaze”−exploring the unseen and multifaceted longterm consequences of severe perineal trauma for women’s health and working lives (Dissertation). Umeå University, Umeå (2025).

[41] Ohlsson-Nevo E Hiyoshi A Norén P Möller M Karlsson J. The Swedish RAND-36: psychometric characteristics and reference data from the mid-Swed health survey. J Patient Rep Outcomes. (2021) 5(1):66. 10.1186/s41687-021-00331-z 34347192 PMC8339183

[42] World Health Organization (WHO), Sweden. Health data overview for the Kingdom of Sweden. (2025). Available online at: https://data.who.int/countries/752 (Accessed December 30, 2025)

[43] Ministry of Health and Social Affairs. Hälso- och sjukvårdslag (2017:30) [Health and Medical Care Act]. (2025). Available online at: https://www.riksdagen.se/sv/dokument-och-lagar/dokument/svensk-forfattningssamling/halso-och-sjukvardslag-201730_sfs-2017-30/ (Accessed December 30, 2025)

[44] The Swedish Social Insurance Agency. Parental benefits. (2025). Available online at: https://www.forsakringskassan.se/english/parents/when-the-child-is-born/parental-benefit (Accessed December 30, 2025)

[45] Forte. Kvinnors hälsa och sjukdomarch—kartläggning och analys av forskningsbehov [Women’s health and diseases—mapping and analysis of research needs]. (2023). Report No.: 978-91-88561-56-5. Available online at: https://forte.se/publikation/kvinnors-halsa-och-sjukdomar-kartlaggning-och-analys-av-forskningsbehov/ (Accessed December 30, 2025)

[46] Fransson E Grönqvist E Iliadis S Lindahl E. Kvinnors hälsa, sjukfrånvaro och inkomster efter barnafödande. Vad vet vi om barneffektens orsaker och vilken roll spelar hälsa och föräldraförsäkringen? [Women’s health, sickness absence and earnings after childbirth. What do we know about the causes of the child effect and what role do health and parental insurance play?]. (2021). Available online at: https://www.ifau.se/Forskning/Publikationer/Rapporter/20212/kvinnors-halsa-sjuk franvaro-och-inkomster-efter-barnafodande/ (Accessed December 30, 2025)

[47] Statistics Sweden. Arbetsmarknadssituationen för befolkningen 15-74 år AKU 2023 [The Labour Market Situation for the Population, 15-74 Years, LFS 2023]. (2023). Available online at: https://www.scb.se/contentassets/45ea8764182045479c8358ce71ab0d93/am0401_2023a01_br_am12sm2401.pdf (Accessed December 30, 2025)

[48] Pajalić Z Pajalić O Saplacan D. Women’s education and profession midwifery in Nordic countries. J Health Sci. (2019) 9(3):127–35. 10.17532/jhsci.2019.820

[49] Socialstyrelsen (National Board of Health and Welfare). Nationella riktlinjer 2025—Graviditet, förlossning och tiden efter (National guidelines 2025—Pregnancy, childbirth and the postnatal period) [Internet]. Stockholm. (2025). Available online at: https://www.socialstyrelsen.se/publikationer/nationella-riktlinjer-2025–graviditet-forlossning-och-tiden-efter–2025-3-9442/ (Accessed December 30, 2025)

[50] Socialstyrelsen [The National Board of Social Affairs and Health]. Nationella riktlinjer 2025—Bäckenbottendysfunktion [National guidelines 2025—Pelvic floor dysfunction]. (2025). Available online at: https://www.socialstyrelsen.se/publikationer/nationella-riktlinjer-2025–backenbottendysfunktion–prioriteringsstod-till-dig-som-beslutar-om-resurser-i-halso–och-sjukvarden–2025-6-9661/ (Accessed December 30, 2025)

[51] Harris PA Taylor R Thielke R Payne J Gonzalez N Conde JG. Research electronic data capture (REDCap)−A metadata-driven methodology and workflow process for providing translational research informatics support. J Biomed Inform. (2009) 42(2):377–81. 10.1016/j.jbi.2008.08.010 18929686 PMC2700030

[52] Harris PA Taylor R Minor BL Elliott V Fernandez M O’Neal L The REDCap consortium: building an international community of software platform partners. J Biomed Inform. (2019) 95:103208. 10.1016/j.jbi.2019.103208 31078660 PMC7254481

[53] Hays R Sherbourne C Mazel R. The rand 36-item health survey 1.0. Health Econ. (1993) 2(3):217–27. 10.1002/hec.4730020305 8275167

[54] Hays RD Morales LS. The RAND-36 measure of health-related quality of life. Ann Med. (2001) 33(5):350–7. 10.3109/07853890109002089 11491194

[55] Orwelius L Nilsson M Nilsson E Wenemark M Walfridsson U Lundstrom M The Swedish RAND-36 health survey−reliability and responsiveness assessed in patient populations using Svensson’s method for paired ordinal data. J Patient Rep Outcomes. (2017) 2(1):4. 10.1186/s41687-018-0030-0 29757320 PMC5934928

[56] Andersen JR Breivik K Engelund IE Iversen MM Kirkeleit J Norekvål TM Correlated physical and mental health composite scores for the RAND-36 and RAND-12 health surveys: can we keep them simple? Health Qual Life Outcomes. (2022) 20(1):89. 10.1186/s12955-022-01992-0 35659237 PMC9166415

[57] van Buuren S. Flexible Imputation of Missing Data. Boca Raton: CRC Press (2012).

[58] RAND Health Care. 36-Item Short Form Survey (SF-36) Scoring Instructions. (2025). Available online at: https://www.rand.org/health-care/surveys_tools/mos/36-item-short-form/scoring.html (Accessed December 30, 2025)

[59] Field AP. Discovering Statistics Using IBM SPSS Statistics. 6th ed. London: Sage Publications (2024).

[60] von Elm E Altman DG Egger M Pocock SJ Gøtzsche PC Vandenbroucke JP The strengthening the reporting of observational studies in epidemiology (STROBE) statement: guidelines for reporting observational studies. J Clin Epidemiol. (2008) 61(4):344–9. 10.1016/j.jclinepi.2007.11.008 18313558

[61] World Medical Association. World medical association declaration of Helsinki: ethical principles for medical research involving human participants. JAMA. (2025) 333(1):71–4. 10.1001/jama.2024.21972 39425955

[62] Priddis H Dahlen H Schmied V. Women’s experiences following severe perineal trauma: a meta-ethnographic synthesis. J Adv Nurs. (2013) 69(4):748–59. 10.1111/jan.12005 23057716

[63] Larsudd-Kåverud J Åkervall S Molin M Nilsson IE Steyerberg EW Milsom I Predicting obstetric anal sphincter injury in the first and second vaginal delivery and after a cesarean delivery: development and validation of an intrapartal model. J Clin Epidemiol. (2025) 183:111782. 10.1016/j.jclinepi.2025.111782 40216339

[64] Posadzki P Pieper D Bajpai R Makaruk H Könsgen N Neuhaus AL Exercise/physical activity and health outcomes: an overview of Cochrane systematic reviews. BMC Public Health. (2020) 20(1):1724. 10.1186/s12889-020-09855-3 33198717 PMC7670795

[65] Sjögren Forss K Stjernberg L. Physical activity patterns among women and men during pregnancy and 8 months postpartum compared to Pre-pregnancy: a longitudinal study. Front. Public Health. (2019) 7:294. 10.3389/fpubh.2019.00294 31750283 PMC6843064

[66] Poyatos-León R García-Hermoso A Sanabria-Martínez G Álvarez-Bueno C Cavero-Redondo I Martínez-Vizcaíno V. Effects of exercise-based interventions on postpartum depression: a meta-analysis of randomized controlled trials. Birth. (2017) 44(3):200–8. 10.1111/birt.12294 28589648

[67] Deprato A Ruchat SM Ali MU Cai C Forte M Gierc M Impact of postpartum physical activity on maternal depression and anxiety: a systematic review and meta-analysis. Br J Sports Med. (2025) 59(8):550–61. 10.1136/bjsports-2024-108478 39500542

[68] Liu N Wang J Chen DD Sun WJ Li P Zhang W. Effects of exercise on pregnancy and postpartum fatigue: a systematic review and meta-analysis. Eur J Obstet Gynecol Reprod Biol. (2020) 253:285–95. 10.1016/j.ejogrb.2020.08.013 32916639

[69] Jones PAT Ruchat SM Khan-Afridi Z Ali MU Matenchuk BA Leonard S Impact of postpartum physical activity on maternal sleep: a systematic review and meta-analysis. Br J Sports Med. (2025) 59(8):576–83. 10.1136/bjsports-2024-108839 40011015

[70] Lesser IA Turgeon S Nienhuis CP Bean C. Examining the role of physical activity on psychological well-being and mental health postpartum. Women Sport Phys Act J. (2023) 31(1):40–9. 10.1123/wspaj.2022-0057

[71] Evans E Falivene C Briffa K Thompson J Henry A. What is the total impact of an obstetric anal sphincter injury? An Australian retrospective study. Int Urogynecol J. (2020) 31(3):557–66. 10.1007/s00192-019-04108-3 31529328 PMC7093361

[72] Arkel E Torell K Rydhög S Rikner Å Neymark Bachmeier H Gutke A Effects of physiotherapy treatment for patients with obstetric anal sphincter rupture: a systematic review. Eur J Physiother. (2017) 19(2):90–6. 10.1080/21679169.2016.1263872

[73] Peralta LR Cotton WG Dudley DA Hardy LL Yager Z Prichard I. Group-based physical activity interventions for postpartum women with children aged 0–5 years old: a systematic review of randomized controlled trials. BMC Womens Health. (2021) 21(1):435. 10.1186/s12905-021-01581-1 34963456 PMC8714424

[74] Hays RD Prince-Embury S Chen H. RAND-36 Health status Inventory. San Antonio, TX: Psychological Corporation (1998).

[75] Rindner L Nordeman L Strömme G Svenningsson I Premberg Å Hange D Prognostic factors for future mental, physical and urogenital health and work ability in women, 45–55 years: a six-year prospective longitudinal cohort study. BMC Womens Health. (2020) 20(1):171. 10.1186/s12905-020-01015-4 32787825 PMC7425146

[76] SBU [The Swedish Agency for Health Technology Assessment and Assessment of Social Services]. Förlossningsbristningar—diagnostik samt erfarenheter av bemötande och information: en systematisk översikt och utvärdering av medicinska, hälsoekonomiska och etiska aspekter [Childbirth tears—diagnosis and experiences of treatment and information: a systematic review and evaluation of medical, health economic and ethical aspects]. (2021). SBU Utvärderar 323. Available online at: https://www.sbu.se/323 (Accessed December 30, 2025)

[77] Althubaiti A. Information bias in health research: definition, pitfalls, and adjustment methods. J Multidiscip Healthc. (2016) 9:211–7. 10.2147/JMDH.S104807 27217764 PMC4862344

[78] Darko EM Kleib M Olson J. Social Media use for research participant recruitment: integrative literature review. J Med Internet Res. (2022) 24(8):e38015. 10.2196/38015 35925655 PMC9389385

[79] Sullivan M Karlsson J Ware JE. The Swedish SF-36 health survey−I. Evaluation of data quality, scaling assumptions, reliability and construct validity across general populations in Sweden. Qual Life Soc Sci Med. (1995) 41(10):1349–58. 10.1016/0277-9536(95)00125-q 8560302

