# Supplementary material for: Beyond the physical: exploring the complexities of Women’s health after severe perineal trauma—a cross-sectional study on predictors of health-related quality of life in Sweden
Source: Front Glob Womens Health. 2026 Feb 12;7:1734365. doi: 10.3389/fgwh.2026.1734365 (PMC12935897; doi:10.3389/fgwh.2026.1734365)
Supplement: Supplementary file 1 [file Datasheet1.pdf]

Supplementary Material

1 Supplementary Data 1

Missing data analysis

Missing mechanism

Univariate Statistics

	N	Mean	Std. Deviation	Missing		No. of Extremes[a]	
				Count	Percent	Low	High
age	220	39.99	7.747	1	.5	0	12
kvarstaende_besv_vas	216	5.22	2.841	5	2.3	0	0
WAI_total_score	197	38.914	7.3966	24	10.9	9	0
Health_change	218	54.47248	21.602220	3	1.4	6	0
Physical_functioning	221	82.77024	19.805698	0	.0	16	0
Role_limitation_physical	221	67.76018	40.573519	0	.0	0	0
Role_limitation_emotional	221	65.38462	40.696157	0	.0	0	0
Pain	221	74.38914	24.896836	0	.0	0	0
Emotional_well_being	221	67.52036	16.663673	0	.0	4	0
Energy_fatigue	221	46.90045	20.259881	0	.0	0	0
Social_functioning	221	73.02036	24.006643	0	.0	0	0
General_health	221	57.76018	20.914875	0	.0	0	0
RAND_36_PCS	221	70.66993	22.068220	0	.0	0	0
RAND_36_MCS	221	63.20645	21.405422	0	.0	0	0
years_with_SPT	221	10.00	8.189	0	.0	0	12
pfiq_7_total_score	217	54.62658	56.937766	4	1.8	0	8
pfdi_20_total_score	216	77.43979	53.071863	5	2.3	0	6
fodelseland	217			4	1.8		
education_2grps	218			3	1.4		
cohabiting	221			0	.0		
tabacco_use	221			0	.0		
phys_activ_comparison	221			0	.0		
wai_fraga_3g___2	221			0	.0		
wai_fraga_3h___2	221			0	.0		
wai_fraga_3e___2	221			0	.0		
parity	221			0	.0		
instrumental_birth	221			0	.0		
degree_of_SPT	221			0	.0		
reop_2grps	220			1	.5		
birth_mode_post_SPT	208			13	5.9		
employment_status_4grps	221			0	.0		
employment_rate	205			16	7.2		
sick_leave_4grps	220			1	.5		

a. Number of cases outside the range (Q1 - 1.5*IQR, Q3 + 1.5*IQR).

Separate Variance t Tests[a]

		age	kvarstaende_besv_vas	WAI_total_score	Health_change	Physical_functioning	Role_limitation_physical	Role_limitation_emotional	Pain	Emotional_well_being	Energy_fatigue	Social_functioning	General_health	RAND_36_PCS	RAND_36_MCS	years_with_SPT	pfiq_7_total_score	pfdi_20_total_score
WAI_total_score	t	1.5	1.1	.	-.7	.1	-.7	-.3	-.9	.8	.8	-.6	.6	-.4	.0	2.5	.9	1.2
	df	31.8	26.4	.	29.1	25.7	29.5	28.8	29.0	29.1	32.1	29.8	31.4	28.2	29.4	34.1	23.1	22.9
	P(2-tail)	.139	.293	.	.501	.926	.500	.738	.391	.433	.454	.573	.550	.711	.994	.019	.370	.256
	# Present	196	193	197	194	197	197	197	197	197	197	197	197	197	197	197	197	197
	# Missing	24	23	0	24	24	24	24	24	24	24	24	24	24	24	24	20	19
	Mean(Present)	40.23	5.30	38.914	54.12371	82.82854	67.13198	65.05922	73.88325	67.82741	47.20812	72.71574	58.02030	70.46602	63.20262	10.37	55.74893	78.59847
	Mean(Missing)	38.04	4.57	.	57.29167	82.29167	72.91667	68.05556	78.54167	65.00000	44.37500	75.52083	55.62500	72.34375	63.23785	6.92	43.57143	65.42607
birth_mode_post_SPT	t	-2.7	-1.2	1.3	3.0	2.0	1.3	1.3	1.5	.6	1.6	1.5	1.7	2.0	1.5	-2.9	-1.0	-.7
	df	12.5	14.1	11.4	13.9	12.6	13.3	13.1	12.8	13.1	14.2	14.1	13.0	13.0	13.4	12.4	13.2	12.8
	P(2-tail)	.020	.265	.233	.009	.070	.200	.218	.154	.545	.143	.153	.106	.071	.159	.013	.315	.519
	# Present	207	203	186	205	208	208	208	208	208	208	208	208	208	208	208	204	203
	# Missing	13	13	11	13	13	13	13	13	13	13	13	13	13	13	13	13	13
	Mean(Present)	39.43	5.17	39.067	55.48780	83.73665	68.75000	66.42628	75.21635	67.72115	47.35577	73.55769	58.49760	71.55015	63.76522	9.33	53.46250	76.63133
	Mean(Missing)	48.92	6.00	36.318	38.46154	67.30769	51.92308	48.71795	61.15385	64.30769	39.61538	64.42308	45.96154	56.58654	54.26603	20.62	72.89377	90.06410
employment_rate	t	-.4	.9	-6.3	-.1	.2	-1.2	-3.0	-3.0	.1	-.6	-3.3	-.2	-1.2	-2.2	.4	1.7	.3
	df	17.5	17.6	23.0	21.9	16.6	18.3	19.8	20.1	16.6	18.9	19.2	18.3	17.9	18.4	18.1	19.8	17.2
	P(2-tail)	.709	.388	.000	.951	.875	.243	.007	.007	.956	.547	.003	.826	.250	.039	.706	.097	.734
	# Present	204	200	185	202	205	205	205	205	205	205	205	205	205	205	205	201	200
	# Missing	16	16	12	16	16	16	16	16	16	16	16	16	16	16	16	16	16
	Mean(Present)	39.94	5.27	38.538	54.45545	82.84011	66.95122	63.65854	73.36585	67.54146	46.70732	71.82927	57.68293	70.21003	62.43415	10.05	56.08466	77.81310
	Mean(Missing)	40.69	4.63	44.708	54.68750	81.87500	78.12500	87.50000	87.50000	67.25000	49.37500	88.28125	58.75000	76.56250	73.10156	9.31	36.30952	72.77344

For each quantitative variable, pairs of groups are formed by indicator variables (present, missing).

a. Indicator variables with less than 5% missing are not displayed.

All **three variables** with missing over **5%** have t-tests with **significant differences** suggesting that the missingness is related to the observed data, indicating **missing at random (MAR)**. **Crosstabulations** (not provided due to space limitation): **differences in groups** can be seen in relation to fodelseland, education_2grps, cohabiting, cohabiting, phys_activ_comparison, wai_fraga_g+h+e, parity, instrumental_birth, degree_of_SPT, reop_2grps, birth_mode_post_SPT, employment_status_4grps, employment_rate, sick_leave_4grps suggesting that the missingness is related to the observed data, indicating **MAR**.

EM Means[a]

age	kvarstaende_besv_vas	WAI_total_score	Health_change	Physical_functioning	Role_limitation_physical	Role_limitation_emotional	Pain	Emotional_well_being	Energy_fatigue	Social_functioning	General_health	RAND_36_PCS	RAND_36_MCS	years_with_SPT	pfiq_7_total_score	pfdi_20_total_score
39.42	5.31	38.728	55.61193	83.08832	66.92308	64.52991	74.37179	67.12821	46.79487	71.98718	57.62821	70.50285	62.61004	9.30	56.03536	78.40421

a. Little's MCAR test: Chi-Square = ,000, DF = 106, Sig. = 1,000

Non-significant **MCAR**-test (**missing completely at random**) indicating **randomness** of missing values.

Conclusion on mechanism

Tests indicate that missing data is **random (MAR or MCAR).**

Missing pattern

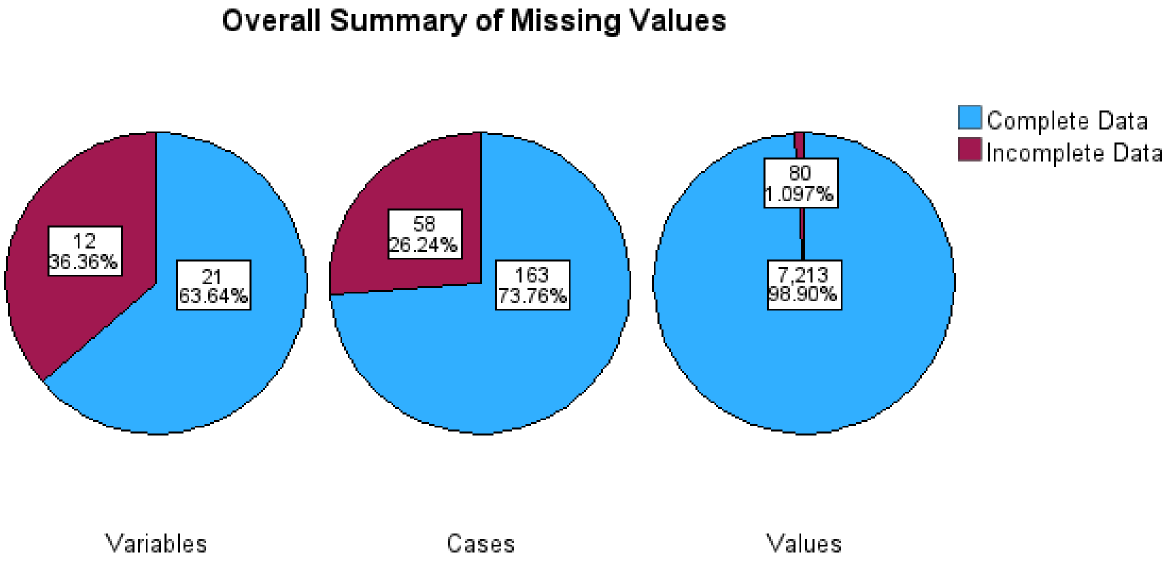

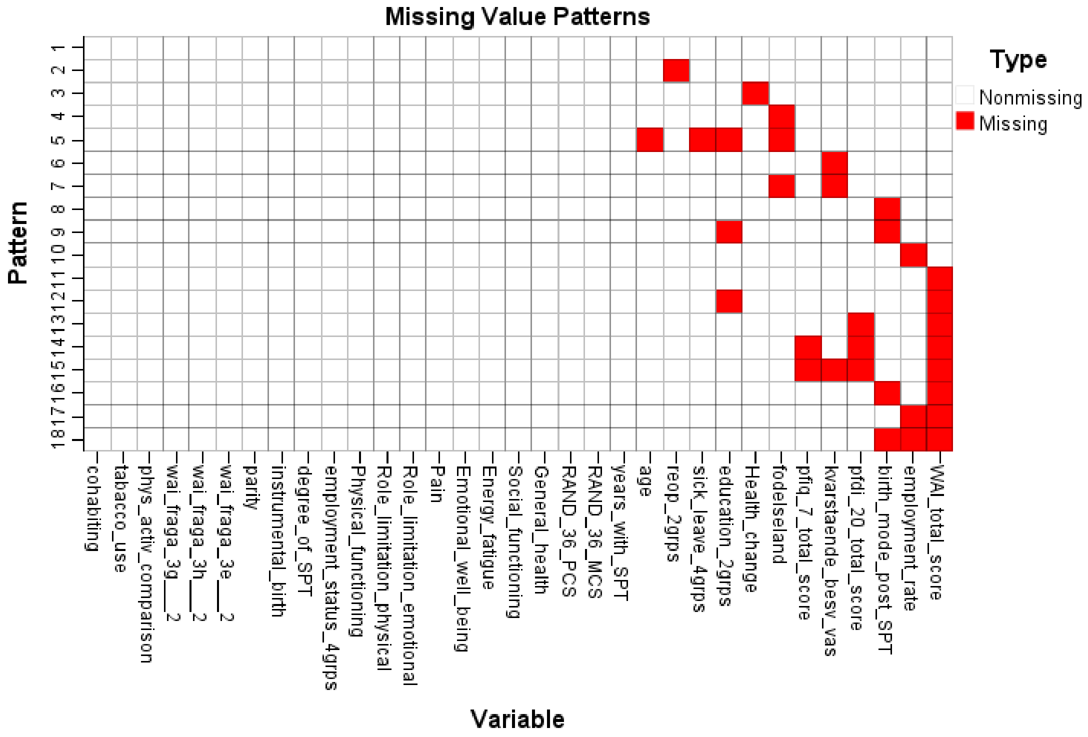

<u>Conclusion on pattern</u>

Non-monotone pattern.

<u>Overall conclusion</u>

There is **randomness** in missing data and a **non-monotone** pattern.

2 Supplementary Figures and Tables

2.1 Supplementary Figures

2.1.1 Supplementary Figure 1

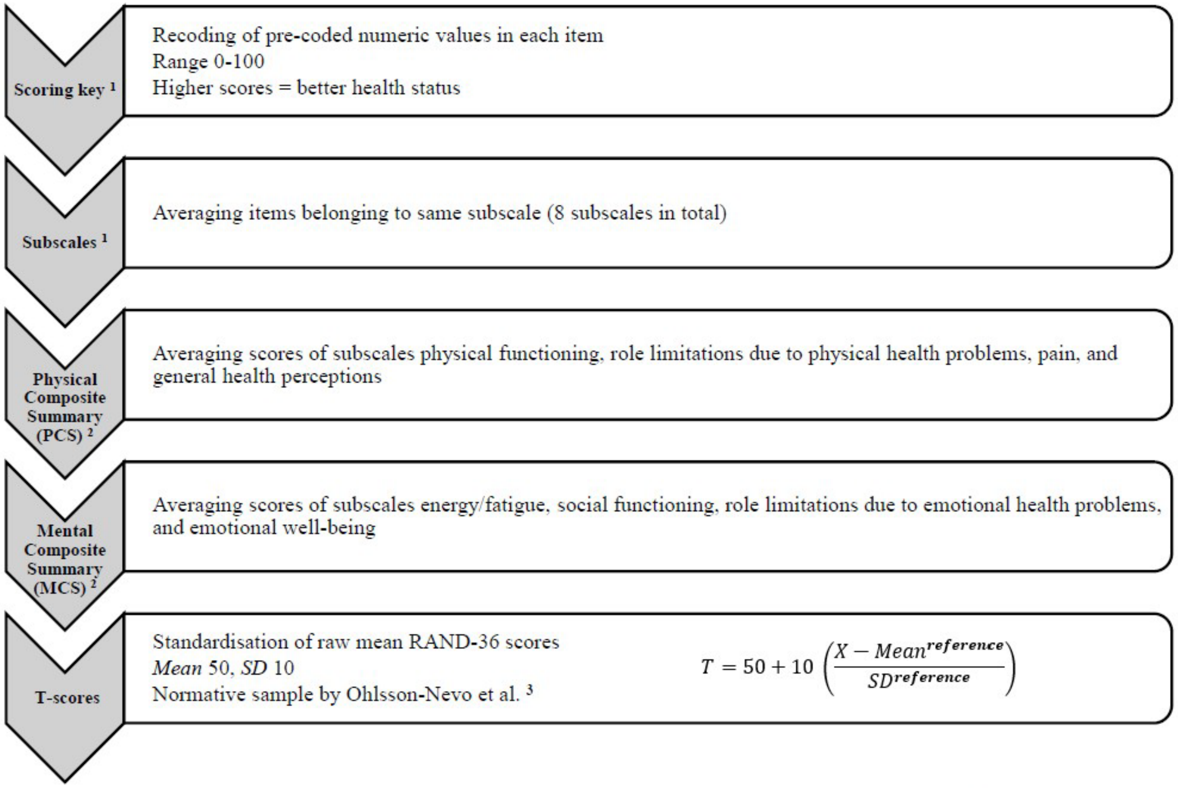

Supplementary Figure 1 Flowchart on scoring procedure of RAND 36-Item Health Survey (Version 1.0) applied in present study

[1] RAND Health Care. 36-Item Short Form Survey (SF-36) Scoring Instructions. (2025) https://www.rand.org/health-care/surveys_tools/mos/36-item-short-form/scoring.html [Accessed December 30, 2025].; [2] Andersen JR, Breivik K, Engelund IE, Iversen MM, Kirkeleit J, Norekvål TM, et al. Correlated physical and mental health composite scores for the RAND-36 and RAND-12 health surveys: can we keep them simple? Health Qual Life Outcomes. (2022) 20(1):89. doi: 10.1186/s12955-022-01992-0.; [3] Ohlsson-Nevo E, Hiyoshi A, Norén P, Möller M, Karlsson J. The Swedish RAND-36: psychometric characteristics and reference data from the Mid-Swed Health Survey. J Patient-Rep Outcomes. (2021) 5(1):66. doi: 10.1186/s41687-021-00331-z.

2.1.2 Supplementary Figure 2

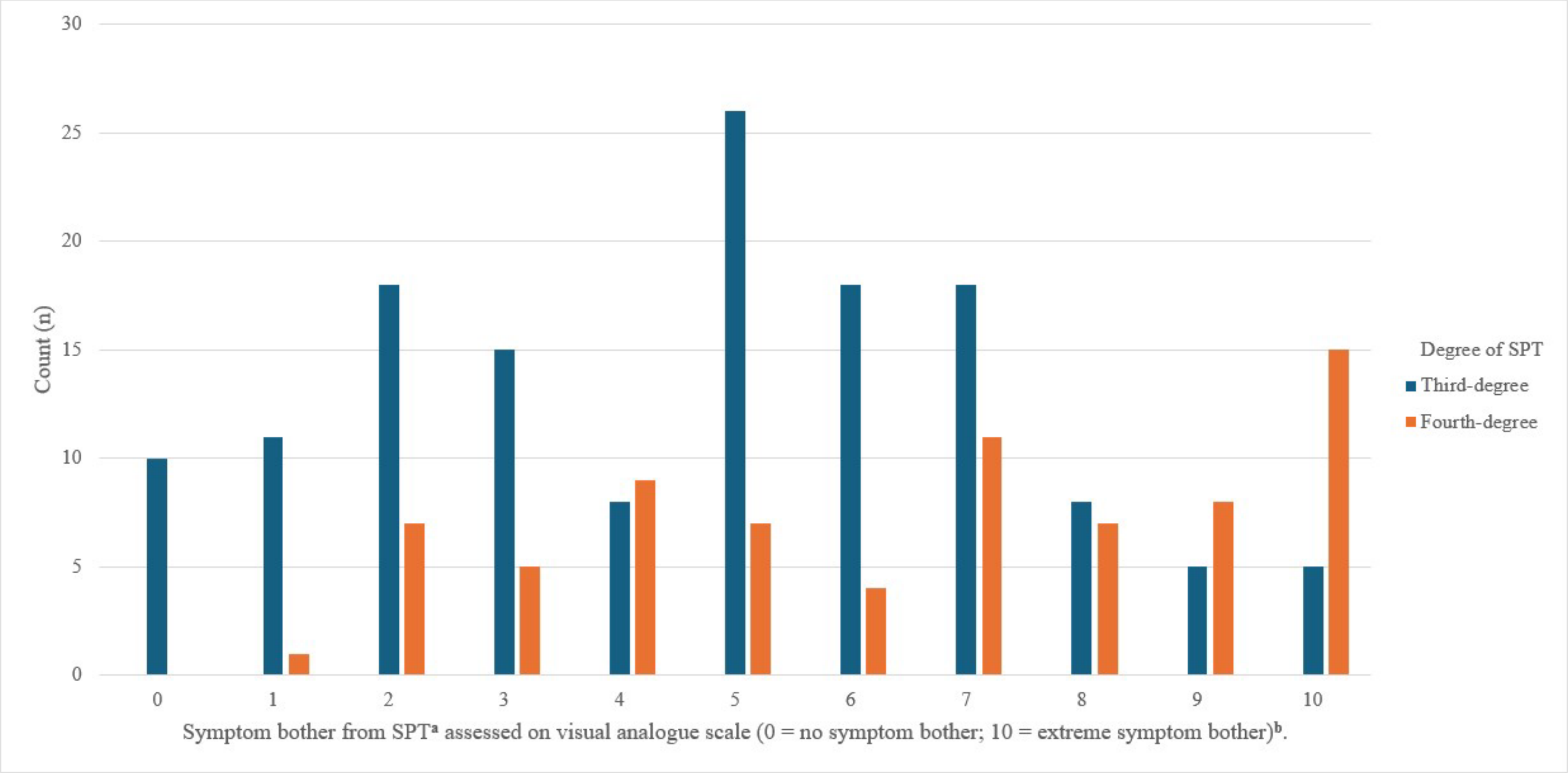

Supplementary Figure 2 Clustered bar chart of symptom bother from SPT by third-degree and fourth-degree SPT [a]

[a] SPT = severe perineal trauma; [b] Cut-off values: mild symptom bother from SPT rated as 0-3 on visual analogue scale; moderate symptom bother from SPT rated as 4-6 on visual analogue scale; severe symptom bother from SPT rated as 7-10 on visual analogue scale.

2.2 Supplementary Tables

2.2.1 Supplementary Table 1 Dropout analysis, total *n* = 253

Outcomes	Included participants (*n* = 221)	Dropouts (*n* = 32)	*p*
**Sociodemographic outcomes**			
Age in years, median	38.00	39.00	0.882 [1]
Missing data, *n*	1	0	
Born in Sweden, n (%)	206 (94.90)	28 (90.30)	0.394 [2]
Missing data, *n*	4	1	
Highest completed educational level			**0.020** [3]
University, *n* (%)	181 (83.00)	21 (65.60)	
Primary or secondary school, *n* (%)	37 (17.00)	11 (34.40)	
Missing data, *n*	3	0	
Co-habiting with partner, n (%)	188 (88.70)	25 (86.2)	0.756 [2]
Missing data, *n*	9	3	
Tobacco user[4], n (%)	30 (13.60)	4 (17.40)	0.539 [2]
Missing data, *n*	0	9	
Level of physical activity compared to before SPT			0.770 [3]
Same level of physical activity, *n* (%)	61 (27.60)	5 (22.70)	
Higher level of physical activity, *n* (%)	38 (17.20)	5 (22.70)	
Lower level of physical activity, *n* (%)	122 (55.20)	12 (54.40)	
Missing data, *n*	0	10	
**Obstetric outcomes**			
Parity			0.708 [2]
Primipara, *n* (%)	53 (24.00)	3 (30.00)	
Multipara, *n* (%)	168 (76.00)	7 (70.00)	

Missing data, *n*	0	22	
Degree of SPT			0.054 [3]
3rd degree, *n* (%)	145 (65.60)	27 (84.40)	
4th degree, *n* (%)	76 (34.40)	5 (15.60)	
Missing data, *n*	0	0	
Number of years living with SPT			0.850 [2]
18 months to 5 years, *n* (%)	64 (29.10)	3 (30.00)	
6 to 10 years, *n* (%)	67 (30.50)	2 (20.00)	
More than 10 years, *n* (%)	89 (40.5)	5 (50.00)	
Missing data, *n*	1	22	
Reconstructive surgery, n (%)	67 (30.50)	2 (22.20)	0.727 [2]
Missing data, *n*	1	23	
**Occupational outcomes**			
Employment status			0.947 [2]
Employed, *n* (%)	193 (87.30)	30 (93.80)	
Self-employed, *n* (%)	8 (3.60)	0	
Student, *n* (%)	10 (4.50)	1 (3.10)	
Other [5], *n* (%)	10 (4.50)	1 (3.10)	
Missing data, *n*	0	0	
Employment rate			0.381 [3]
Full-time (100%), *n* (%)	149 (74.5)	16 (64.00)	
Part-time (75% or more), *n* (%)	40 (20.00)	8 (32.00)	
Part-time (less than 75%), *n* (%)	11 (5.50)	1 (4.00)	
Missing data, *n*	21	7	
History of sick leave in adult life	99 (45.00)	1 (14.30)	0.138 [2]
Missing data, *n*	1	25	

Data presented as number (column percentage), unless otherwise indicated.
[1] Mann-Whitney U-test; [2] Fisher's Exact Test; [3] Chi-Squared test; [4] cigarettes, cigarillos, pipes, e-cigarettes, joints, or snuff; [5] unemployed, sick cash benefit, homemaker, other

2.2.2 Supplementary Table 2 STROBE Statement − Checklist of items that should be included in reports of cross-sectional studies

	Item No	Recommendation	Page No
**Title and abstract**	1	(*a*) Indicate the study's design with a commonly used term in the title or the abstract	p. 1
		(*b*) Provide in the abstract an informative and balanced summary of what was done and what was found	pp. 1−2
**Introduction**			
Background/rationale	2	Explain the scientific background and rationale for the investigation being reported	p. 2
Objectives	3	State specific objectives, including any prespecified hypotheses	p. 2
**Methods**			
Study design	4	Present key elements of study design early in the paper	pp. 2−3
Setting	5	Describe the setting, locations, and relevant dates, including periods of recruitment, exposure, follow-up, and data collection	pp. 3−4
Participants	6	(*a*) Give the eligibility criteria, and the sources and methods of selection of participants	p. 3
Variables	7	Clearly define all outcomes, exposures, predictors, potential confounders, and effect modifiers. Give diagnostic criteria, if applicable	pp. 4−6, Table 1
Data sources/ measurement	8*	For each variable of interest, give sources of data and details of methods of assessment (measurement). Describe comparability of assessment methods if there is more than one group	pp. 4−6, Table 1, Suppl. Figure 1 + 2
Bias	9	Describe any efforts to address potential sources of bias	pp. 9−10
Study size	10	Explain how the study size was arrived at	p. 6, Figure 2
Quantitative variables	11	Explain how quantitative variables were handled in the analyses. If applicable, describe which groupings were chosen and why	pp. 4−6, Table 1, Suppl. Figure 2
Statistical methods	12	(*a*) Describe all statistical methods, including those used to control for confounding	p. 5
		(*b*) Describe any methods used to examine subgroups and interactions	p. 5
		(*c*) Explain how missing data were addressed	p. 5, Suppl. Data 1

		(*d*) If applicable, describe analytical methods taking account of sampling strategy	pp. 3−5, Figure 2
		(*e*) Describe any sensitivity analyses	p. 5, Table 5
**Results**			
Participants	13*	(a) Report numbers of individuals at each stage of study − e.g. numbers potentially eligible, examined for eligibility, confirmed eligible, included in the study, completing follow-up, and analyzed	p. 6, Figure 2
		(b) Give reasons for non-participation at each stage	Figure 2
		(c) Consider use of a flow diagram	Figure 2
Descriptive data	14*	(a) Give characteristics of study participants (e.g. demographic, clinical, social) and information on exposures and potential confounders	p. 6, Table 2
		(b) Indicate number of participants with missing data for each variable of interest	Table 2
Outcome data	15*	Report numbers of outcome events or summary measures	pp. 6−7, Table 3
Main results	16	(*a*) Give unadjusted estimates and, if applicable, confounder-adjusted estimates and their precision (e.g., 95% confidence interval). Make clear which confounders were adjusted for and why they were included	p. 7, Table 4 + 5
		(*b*) Report category boundaries when continuous variables were categorized	N/A
		(*c*) If relevant, consider translating estimates of relative risk into absolute risk for a meaningful time period	N/A
Other analyses	17	Report other analyses done − e.g. analyses of subgroups and interactions, and sensitivity analyses	pp. 6−7, Table 3 + 4 + 5, Suppl. Table 1
**Discussion**			
Key results	18	Summarize key results with reference to study objectives	pp. 7−8
Limitations	19	Discuss limitations of the study, taking into account sources of potential bias or imprecision. Discuss both direction and magnitude of any potential bias	pp. 9−10
Interpretation	20	Give a cautious overall interpretation of results considering objectives, limitations, multiplicity of analyses, results from similar studies, and other relevant evidence	pp. 10−11
Generalizability	21	Discuss the generalizability (external validity) of the study results	pp. 9−10
**Other information**			

Funding	22	Give the source of funding and the role of the funders for the present study and, if applicable, for the original study on which the present article is based	p. 11

*Give information separately for cases and controls in case-control studies and, if applicable, for exposed and unexposed groups in cohort and cross-sectional studies.

Note: An Explanation and Elaboration article discusses each checklist item and gives methodological background and published examples of transparent reporting. The STROBE checklist is best used in conjunction with this article (freely available on the Web sites of PLoS Medicine at http://www.plosmedicine.org/, Annals of Internal Medicine at http://www.annals.org/, and Epidemiology at http://www.epidem.com/). Information on the STROBE Initiative is available at www.strobe-statement.org.

Reference: von Elm E, Altman DG, Egger M, Pocock SJ, Gøtzsche PC, Vandenbroucke JP. STROBE Initiative. The Strengthening the Reporting of Observational Studies in Epidemiology (STROBE) statement: guidelines for reporting observational studies. J Clin Epidemiol. (2008) 61(4):344-9. doi: 10.1016/j.jclinepi.2007.11.008.
